# Supplementary material for: High-dimensional non-Abelian holonomy in integrated photonics
Source: Nat Commun. 2025 Apr 17;16:3650. doi: 10.1038/s41467-025-58794-3 (PMC12006547; doi:10.1038/s41467-025-58794-3)
Supplement: Supplementary file 1 — Supplementary information [file 41467_2025_58794_MOESM1_ESM.pdf]

Supplementary Information

High-dimensional non-Abelian holonomy in integrated photonics

Youlve Chen[1,2], Yunru Fan[3,4], Larsonneur Gulliver[1], Jinlong Xiang[1], An He[1], Guohuai Wang[2], Xu-Lin Zhang[2*], Guancong Ma[5], Qiang Zhou[3,4], Guangcan Guo[3,4], Yikai Su[1*], and Xuhan Guo[1*]

[1]State Key Laboratory of Photonics and Communications, School of Information and Electronic Engineering, Shanghai Jiao Tong University; Shanghai, 200240, China.

[2]State Key Laboratory of Integrated Optoelectronics, College of Electronic Science and Engineering, Jilin University, Changchun, China.

[3]Institute of Fundamental and Frontier Sciences, University of Electronic Science and Technology of China, Chengdu 611731, China

[4]Center for Quantum Internet, Tianfu Jiangxi Laboratory, Chengdu 641419, China

[5]Department of Physics, Hong Kong Baptist University, Kowloon Tong, Hong Kong, China.

*Corresponding author Email: xulin_zhang@jlu.edu.cn; yikaisu@sjtu.edu.cn; guoxuhan@sjtu.edu.cn

This Supplementary Information contains 4 Supplementary Notes, 34 Supplementary Figures and 5 References.

Supplementary Note 1. Theory

We consider a time-dependent quantum system described by a Hamiltonian. The instantaneous eigenfunction can be written as:

$$H(t)|\varphi_n(t)\rangle = E_n(t)|\varphi_n(t)\rangle \tag{1}$$

The corresponding Schrödinger equation can be written as:

$$i\frac{\partial}{\partial t}|\varphi(t)\rangle = H(t)|\varphi(t)\rangle \tag{2}$$

where

$$|\varphi(t)\rangle = \sum a_n(t)|n(t)\rangle \mathrm{e}^{-\mathrm{i}\int_0^t E_n(\tau)d\tau} \tag{3}$$

And, $a_n(t)$ is the instantaneous probability amplitude of eigenstate $|n\rangle$. Substitute into Equation (2), and left multiplication $|n\rangle$, we can get:

$$\dot{a}_n(t) = -a_n(t)\langle n(t) \mid \dot{n}(t)\rangle - \sum_{m\neq n} a_m(t)\mathrm{e}^{-\mathrm{i}\int_0^t E_n(\tau)-E_m(\tau)d\tau}\langle n(t) \mid \dot{m}(t)\rangle \tag{4}$$

If we add the dynamic phase to the instantaneous probability amplitude, that is:

$$a_n'(t) = a_n(t)\mathrm{e}^{-\mathrm{i}\int_0^t E_n(\tau)d\tau} \tag{5}$$

Substitute into Equation (4),

$$\dot{a}_n'(t) = -a_n'(t)\langle n(t) \mid \dot{n}(t)\rangle - iE_n(t)a_n'(t) - \sum_{m\neq n} a_m'(t)\langle n(t) \mid \dot{m}(t)\rangle \tag{6}$$

For the non-degenerate case, that satisfies:

$$\beta = \left|\frac{\langle n(t) \mid \dot{m}(t)\rangle}{E_n(t) - E_m(t)}\right| \ll 1 \tag{7}$$

We can derive that:

$$a_n'(t) = \mathrm{e}^{-\mathrm{i}\int_0^t E_n(\tau)d\tau - \int_0^t \langle n(\tau)|\dot{n}(\tau)\rangle d\tau} \tag{8}$$

The adiabatic phase:

$$\gamma(t) = \mathrm{i}\int_0^t \langle n(\tau) \mid \dot{n}(\tau)\rangle d\tau \tag{9}$$

is the well-known Pancharatnam-Berry phase, it's the one-dimension Abelian geometric phase and cannot be removed by gauge transformation.

For the degenerate case, we consider an m-fold degenerate case:

$$H(t)|\varphi_{na}(t)\rangle = E_n(t)|\varphi_{na}(t)\rangle, a = 1,2, \dots m \tag{10}$$

The final state and initial state can be connected by $U(m)$ after the evolution:

$$|\varphi_{na}(T)\rangle = \sum_{b=1}^{m} U_{ab}|\varphi_{nb}(0)\rangle \tag{11}$$

Where,

$$U(\gamma)=P\ \exp(\mathrm{i}\oint_{\gamma}A) \tag{12}$$

With A is the Wilczek-Zee connection:

$$A_{ab} = \mathrm{i}\langle\varphi_{na} \mid \dot{\varphi}_{nb}\rangle \tag{13}$$

Basic four-site Hamiltonian

Now we consider the basic four-site Hamiltonian in the main text reproduced by the four-waveguide system, which reads:

$$H(z) = \begin{bmatrix} \beta & \kappa_{\mathrm{AX}}(z) & \kappa_{\mathrm{BX}}(z) & \kappa_{\mathrm{CX}}(z) \\ \kappa_{\mathrm{AX}}(z) & \beta & 0 & 0 \\ \kappa_{\mathrm{BX}}(z) & 0 & \beta & 0 \\ \kappa_{\mathrm{CX}}(z) & 0 & 0 & \beta \end{bmatrix} \tag{14}$$

This Hamiltonian supports two degenerate modes, the eigenvalues are β, where β is the effective index of the individual waveguide. These two degenerate modes are called "dark mode" [1]. Their mode profile can be written as:

$$\begin{aligned} |D_1\rangle &= \sin\ \varphi|B\rangle - \cos\ \varphi|C\rangle \\ |D_2\rangle &= \cos\ \eta|A\rangle - \cos\ \varphi \sin\ \eta|B\rangle - \sin\ \varphi \sin\ \eta|C\rangle \end{aligned} \tag{15}$$

This Hamiltonian also supports two non-degenerate modes, which are called "bright mode"[2], the eigenmodes and eigenvalues are written as:

$$\begin{aligned} |B_1\rangle &= \frac{1}{\sqrt{2}}|X\rangle + \frac{1}{\sqrt{2}}\sin\ (\eta)|A\rangle + \frac{1}{\sqrt{2}}\cos\ (\eta)\sin\ \varphi|B\rangle + \frac{1}{\sqrt{2}}\cos\ (\eta)\cos\ \varphi|C\rangle \\ E_1 &= \beta + \sqrt{\kappa_{\mathrm{AX}}^2 + \kappa_{\mathrm{BX}}^2 + \kappa_{\mathrm{CX}}^2} \end{aligned} \tag{16}$$

$$\begin{aligned} |B_2\rangle &= -\frac{1}{\sqrt{2}}|X\rangle + \frac{1}{\sqrt{2}}\sin\ (\eta)|A\rangle + \frac{1}{\sqrt{2}}\cos\ (\eta)\sin\ \varphi|B\rangle \\ &\quad + \frac{1}{\sqrt{2}}\cos\ (\eta)\cos\ \varphi|C\rangle \\ E_2 &= \beta - \sqrt{\kappa_{\mathrm{AX}}^2 + \kappa_{\mathrm{BX}}^2 + \kappa_{\mathrm{CX}}^2} \end{aligned} \tag{17}$$

Where, $\varphi=\arctan(\kappa_{\mathrm{CX}}/\kappa_{\mathrm{BX}})$, $\eta=\arctan(\kappa_{\mathrm{AX}}/\sqrt{\kappa_{\mathrm{BX}}^2 + \kappa_{\mathrm{CX}}^2})$.

So the coupling coefficients can be normalized as the $\boldsymbol{\kappa}$ sphere (Fig. 1a in the main text):

$$\begin{aligned} \kappa_{\mathrm{AX}} &= \kappa \sin\ (\eta) \\ \kappa_{\mathrm{BX}} &= \kappa \cos\ (\eta)\cos\ (\varphi) \\ \kappa_{\mathrm{CX}} &= \kappa \cos\ (\eta)\sin\ (\varphi) \end{aligned} \tag{18}$$

Where, $\kappa = \sqrt{\kappa_{\mathrm{AX}}^2 + \kappa_{\mathrm{BX}}^2 + \kappa_{\mathrm{CX}}^2}$.

It's straightforward that:

$$\langle D_1 \mid \dot{D}_1\rangle = 0 \tag{19}$$

$$\langle D_2 \mid \dot{D}_2 \rangle = 0$$

The mode in degenerate subspace can be viewed as the superposition of $|D_1\rangle$ and $|D_2\rangle$, that is:

$$|\varphi(z)\rangle = a_1'(t)|D_1\rangle + a_2'(t)|D_2\rangle \tag{20}$$

When the coupling coefficient evolves adiabatically in a holonomy, the modes remain in the degenerate subspace. Substitute Equation (19) into Equation (6), we can get:

$$\begin{aligned} \dot{a}_1'(z) &= -a_2'(z)\langle D_1(z) \mid \dot{D}_2(z)\rangle \\ \dot{a}_2'(z) &= -a_1'(z)\langle D_2(z) \mid \dot{D}_1(z)\rangle \end{aligned} \tag{21}$$

Thus, arbitrary input that excites degenerate modes can evolve as a unitary matrix. The differential and integral of *z* can be substituted with the differential and integral of parameters in Hilbert space. The unitary matrix is the curve integrals of the Wilczek-Zee connection:

$$U(\gamma) = P\exp\,(\mathrm{i}\oint_\gamma A) \tag{22}$$

Where, P is the path-ordering operator and A is the non-Abelian connection, which reads:

$$\left(A_{\varphi,\eta}\right)_{ij} = \mathrm{i}\langle D_i|\partial_{\varphi,\eta}|D_j\rangle \tag{23}$$

Where $|D_i\rangle$, $|D_j\rangle$ are degenerate modes in Equation (15), we can get:

$$A_\varphi = \mathrm{i}\begin{bmatrix} 0 & \sin\,\eta \\ -\sin\,\eta & 0 \end{bmatrix}, A_\eta = 0 \tag{24}$$

Substitute into Equation (22), we can get:

$$U(\gamma) = \exp\,(\mathrm{i}\oint_\gamma \sin\eta d\varphi \cdot -\sigma_y) = \exp\left(-\mathrm{i}\theta\sigma_y\right) \tag{25}$$

$$\begin{bmatrix} |D_1\rangle \\ |D_2\rangle \end{bmatrix}_{(\text{final})} = \begin{bmatrix} \cos\,\theta & -\sin\,\theta \\ \sin\,\theta & \cos\,\theta \end{bmatrix}\begin{bmatrix} |D_1\rangle \\ |D_2\rangle \end{bmatrix}_{(\text{initial})} \tag{26}$$

The input and output ports are waveguides B and C. At the input (output) port, the light can be viewed as the superposition of the degenerate mode $|D_1\rangle$and $|D_2\rangle$from Equation (15).

$$\begin{bmatrix} |B\rangle \\ |C\rangle \end{bmatrix} = \frac{1}{\sqrt{2}}\begin{bmatrix} 1 & -1 \\ -1 & -1 \end{bmatrix}\begin{bmatrix} |D_1\rangle \\ |D_2\rangle \end{bmatrix} \tag{27}$$

Assume that

$$S = \frac{1}{\sqrt{2}}\begin{bmatrix} 1 & -1 \\ -1 & -1 \end{bmatrix} \tag{28}$$

Substitute into Equation (22), we can get

$$\begin{bmatrix}|B\rangle \\ |C\rangle\end{bmatrix}_{\text{(final)}} = S^{-1}US\begin{bmatrix}|B\rangle \\ |C\rangle\end{bmatrix}_{\text{(initial)}} = \begin{bmatrix}\cos\theta & \sin\theta \\ -\sin\theta & \cos\theta\end{bmatrix}\begin{bmatrix}|B\rangle \\ |C\rangle\end{bmatrix}_{\text{(initial)}}$$

$$= \exp\left(\mathrm{i}\theta\sigma_y\right)\begin{bmatrix}|B\rangle \\ |C\rangle\end{bmatrix}_{\text{(initial)}} \tag{29}$$

Therefore, the input and output waveguides can be connected by a unitary matrix $\exp\left(\mathrm{i}\theta\sigma_{\mathrm{y}}\right)$.

For the two-mode braiding, there is a flip relation[2] between the input /output port and degenerate modes, that is,

$$\begin{bmatrix}|B\rangle \\ |C\rangle\end{bmatrix}_{\text{(initial)}} = \begin{bmatrix}1 & 0 \\ 0 & -1\end{bmatrix}\begin{bmatrix}|D_1\rangle \\ |D_2\rangle\end{bmatrix}_{\text{(initial)}}$$

$$\begin{bmatrix}|B\rangle \\ |C\rangle\end{bmatrix}_{\text{(final)}} = \begin{bmatrix}0 & -1 \\ -1 & 0\end{bmatrix}\begin{bmatrix}|D_1\rangle \\ |D_2\rangle\end{bmatrix}_{\text{(final)}} \tag{30}$$

Where,

$$\begin{bmatrix}|D_1\rangle \\ |D_2\rangle\end{bmatrix}_{\text{(final)}} = \begin{bmatrix}1 & 0 \\ 0 & 1\end{bmatrix}\begin{bmatrix}|D_1\rangle \\ |D_2\rangle\end{bmatrix}_{\text{(initial)}} \tag{31}$$

This is because the curve integral (excluding the dashed line) of Supplementary Fig. 10d is zero.

Then we can get:

$$\begin{bmatrix}|B\rangle \\ |C\rangle\end{bmatrix}_{\text{(final)}} = \begin{bmatrix}0 & 1 \\ -1 & 0\end{bmatrix}\begin{bmatrix}|B\rangle \\ |C\rangle\end{bmatrix}_{\text{(initial)}} \tag{32}$$

It is equivalently to the curve integral (including the dashed line) of Supplementary Fig. 10d, that is, $\pi/2$.

Supplementary Note 2. Wavelength sensitivity analysis and undesirable factors

Theory

In theory, the geometric phase is relatively stable to wavelength variation, due to the evolution path in parameter Hilbert space (φ, η, which are quotients of different κ) is insensitive to wavelength regardless of fluctuations of κ-wavelength relation.

The coupling coefficient κ is dependent on the evanescent of guide mode. Assume that the mode is well-confined on waveguides, κ can be approximately written as[3]:

$$\kappa = \frac{2h^2 q e^{-qs}}{k_0^2 \beta W (q^2 + h^2)} (\frac{2\pi}{\lambda}) (n_{\text{SiN}}^2 - n_{\text{SiO}_2}^2) \tag{33}$$

Where W is the width of waveguides, s is the separation, q is the decay rate of the evanescent wave, h and β are propagation constant in y and z directions,

$$q = \sqrt{\beta^2 - k_0^2 n_{\text{SiO}_2}^2} \tag{34}$$

So we can get

$$\kappa_{\text{BX}} = \frac{2h^2 q e^{-q g_{\text{BX}}}}{k_0^2 \beta W (q^2 + h^2)} (\frac{2\pi}{\lambda}) (n_{\text{SiN}}^2 - n_{\text{SiO}_2}^2),$$
$$\kappa_{\text{CX}} = \frac{2h^2 q e^{-q g_{\text{CX}}}}{k_0^2 \beta W (q^2 + h^2)} (\frac{2\pi}{\lambda}) (n_{\text{SiN}}^2 - n_{\text{SiO}_2}^2) \tag{35}$$

$$\frac{\kappa_{\text{BX}}}{\kappa_{\text{CX}}} = \mathrm{e}^{-q(g_{\text{BX}} - g_{\text{CX}})} \tag{36}$$

Now we come to the first parameter, φ:

$$\varphi(\lambda) = \arctan\left(\frac{\kappa_{\text{BX}}}{\kappa_{\text{CX}}}\right) = \arctan\left(\mathrm{e}^{-q(g_{\text{BX}} - g_{\text{CX}})}\right) \tag{37}$$

The wavelength-dependent term is evanescent wave decay rate q, which is relevant to the dispersion relation.

$$\varphi(\lambda) = \arctan\left(\mathrm{e}^{-q(\lambda)(g_{\text{BX}} - g_{\text{CX}})}\right) \tag{38}$$

Take the derivative of λ,

$$\frac{d\varphi(\lambda)}{d\lambda} = \frac{-(g_{\text{BX}} - g_{\text{CX}})\dot{q}(\lambda)}{1 + \mathrm{e}^{-2q(\lambda)(g_{\text{BX}} - g_{\text{CX}})}} \tag{39}$$

We assume $g_{\text{BX}} \leq g_{\text{CX}}$ (the case that $g_{\text{BX}} \geq g_{\text{CX}}$ is the mirror symmetry of $\varphi = \pi/2$). If $g_{\text{BX}} = g_{\text{CX}}$, $\dot{\varphi}(\lambda) = 0$, it's flat-band. If $g_{\text{BX}} << g_{\text{CX}}$, $\dot{\varphi}(\lambda) \to 0$, $\varphi(\lambda) \to \pi/2$, it's also flat-band. It can be observed in Supplementary Fig. 4. For other cases, we can also see that φ varies less than 0.1 rad in a range of wavelength from 1300 nm to 1500 nm.

Now we come to another parameter, sin(η):

$$\eta = \arctan \frac{\kappa_{\mathrm{AX}}}{\sqrt{\kappa_{\mathrm{BX}}^2 + \kappa_{\mathrm{CX}}^2}} = \arctan \frac{\frac{\kappa_{\mathrm{AX}}}{\kappa_{\mathrm{CX}}}}{\sqrt{\frac{\kappa_{\mathrm{BX}}^2}{\kappa_{\mathrm{CX}}^2} + 1}}$$

$$= \arctan \frac{W \mathrm{e}^{-q(g_{\mathrm{AX}} - g_{\mathrm{CX}})}}{H\sqrt{\mathrm{e}^{-2q(g_{\mathrm{BX}} - g_{\mathrm{CX}})} + 1}} \tag{40}$$

Where W is the width of the waveguide, H is the height of the waveguide.

If $\kappa_{\mathrm{BX}} << \kappa_{\mathrm{CX}}$, $\kappa_{\mathrm{BX}} >> \kappa_{\mathrm{CX}}$ or $\kappa_{\mathrm{BX}} = \kappa_{\mathrm{CX}}$, the above equation can be simplified as,

$$\eta = \arctan \frac{\kappa_{\mathrm{AX}}}{\kappa_{\mathrm{CX}}}, \eta = \arctan \frac{\kappa_{\mathrm{AX}}}{\kappa_{\mathrm{BX}}} \text{ or } \eta = \arctan \frac{\kappa_{\mathrm{AX}}}{\sqrt{2}\kappa_{\mathrm{BX}}} \tag{41}$$

Which is the quotient of two κ similar to φ that we have discussed above, it's flat-band.

For other cases, we select different g_{BX} and g_{CX} to calculate $\sin(\eta)$ from 1300 nm to 1500 nm (the thickness of inter-layer silica is fixed to 650 nm), which is shown in Supplementary Fig. 4. We can also see that $\sin(\eta)$ varies less than 0.1.

Actual device

From the discussion above, we can observe the parameters are stable at a wavelength of 1300-1500 nm in theory. However, actual devices face some problems: The first issue is that, at short wavelength, due to the relatively small coupling coefficient, the diabatic effect will increase and some power will jump to the bright-mode subspace, further affecting the precise of unitary matrices (adiabatic requires $\frac{\langle B_{1,2} | \partial_z D \rangle}{|\beta_D - \beta_{B_{1,2}}|} \ll 1$, small κ results in small $|\beta_{|D\rangle}$-$\beta_{|B_{1,2}\rangle}|$). Thus, in simulation we use a mode expansion monitor to monitor the power of bright modes, ensuring that more than 90% of power is in degenerate subspace.

The second issue is that, at long wavelength, non-perfect degeneracy will affect the precision of unitary matrices. This effect is remarkable especially for unitary matrices with large θ, which experience a small gap at step 2. As shown in Supplementary Fig. 5, the four modes are solved through Lumerical Mode Solutions, we can observe that at small g_{BX} and g_{CX}, the n_{eff} of two degenerate modes will bifurcate. The increased n_{eff} of $|D_1\rangle$ is due to the self-coupling effect[4], that is, high-refractive-index SiN waveguide X has large overlap area with $|D_1\rangle$. The decrease n_{eff} of $|D_2\rangle$ is due to the unwanted κ such as κ_{AB} and κ_{AC} can not be negligible, which results in the decrease of the eigenvalue of $|D_2\rangle$. Thus, we reconsider Equation (6), and rewrite Equation (21) as:

$$\begin{aligned} \dot{a}_1'(z) &= \frac{\delta}{2} a_1'(z) - a_2'(z) \langle D_1(z) \mid \dot{D}_2(z) \rangle \\ \dot{a}_2'(z) &= -\frac{\delta}{2} a_2'(z) - a_1'(z) \langle D_2(z) \mid \dot{D}_1(z) \rangle \end{aligned} \tag{42}$$

So the non-Abelian connection in Equation (24) can be rewritten as:

$$A = \mathrm{i}\begin{bmatrix} \frac{\delta}{2}\left(\frac{\partial \varphi, \eta}{\partial z}\right)^{-1} & \sin\,\eta \\ -\sin\,\eta & -\frac{\delta}{2}\left(\frac{\partial \varphi, \eta}{\partial z}\right)^{-1} \end{bmatrix} \tag{43}$$

Where δ is the effective index bifurcate between $|D_1\rangle$and $|D_2\rangle$. This term will hinder the transformation of degenerate modes through non-Abelian connection sin(η), the mode will turn to stay in itself, effectively enlarging the enclosure in κ sphere, leading to a larger θ. So some optimizations are needed.

One method is to decrease the $(\frac{\partial\varphi,\eta}{\partial z})^{-1}$, which means accelerating the speed of parameter variation such as appropriately reducing the length of the device when going through the severe degeneracy-broken region (Note that adiabatic condition should be maintained). The second method is increasing the inter-layer silica thickness h to decrease unwanted κ_{AB} and κ_{AC}, but at the cost of longer device length. The third method is to optimize the path in parameter Hilbert space, such as bypassing the region with serious degeneracy-broken as Supplementary Fig. 5, if the $|\beta_{|D_1\rangle}$-$\beta_{|D_2\rangle}|$ is at the order of 10^{-4}, the influence of non-perfect degeneracy is small.

In the actual fabrication process, some voids will be generated at a small gap due to the limited filling gap capability of PECVD. Vertical etching angles will increase the difficulty of filling the gap. So the etching angle is around 82° as Supplementary Fig. 5e. Appropriate void generated in PECVD can suppress the degeneracy-broken due to the low refractive index of air can suppress unwanted κ_{AB}, κ_{AC} and can reduce the self-coupling effect.

Supplementary Note 3. Planarization for the second layer

After fabricating the SiN waveguides on layer 1, silica is covered on the surface of the SiN waveguides through PECVD. However, the surface of silica is very uneven due to the existing structure of layer 1 waveguides (Supplementary Fig. 15a). Ideally, CMP (chemical mechanical polishing) technology is needed to planarize the silica, otherwise the SiN waveguide on layer 2 will be twisted and deformed (Supplementary Fig. 15b). However, due to the limitation of equipment and the lack of CMP with high precision, we use an alternative method to make the silica flat. We add some supporting waveguides, utilizing the void generated by the deficient filling gap capability of PECVD, early closure point can be observed (Supplementary Fig. 15c) and can generate a relatively flat surface for layer 2 (Supplementary Fig. 15d-e). Note that these auxiliary subwavelength supporting structures are simulated with negligible impact on the main waveguides due to the large n_{eff} mismatch. Supplementary Fig. 15f,h shows SEM graphs of the main waveguides and supporting waveguides on layer 1, Supplementary Fig. 15g,i shows SEM graphs of the main waveguides on layer 2, we can observe that the waveguides on layer 2 are nearly not deformed.

Supplementary Note 4. Phase information

Here we will discuss the detection of phase information through the interference method. The first is a two-order unitary matrix. As Supplementary Fig. 22 shows, at the output port, we use a combiner to merge the two beams of output light. These two beams will interfere with each other, and the phase difference will be shown in the transmission T. In theory, the phase difference should be 0 or π. As Supplementary Fig. 22a shows, if the light is injected from B, there is a phase difference π between B and C at the output position, and the destructive interference will be shown from the output transmission T_{des}; if the light is injected from C, there is no phase difference between B and C at the output position, and the constructive interference will be shown from the output transmission T_{cons}. The theoretical T_{cons}, T_{des} are shown in Supplementary Fig. 22a. Note that the amplitude of the two beams is generally different, and the transmission energy of the interference is not simply the superposition of the two beams of light. This is because the merged waveguide can only transmit the fundamental mode TE_0, thus we could not simply add or subtract the amplitudes of the two beams but need to calculate the symmetric component of the two beams of light before the interference. Only the energy of the symmetric component will remain, and the energy of the anti-symmetric component will be dissipated. Because the waveguide can only transmit TE_0 mode after the combiner. The experimental transmission spectra of constructive and destructive are shown in Supplementary Fig. 22d, $T_{\mathrm{cons}} > T_{\mathrm{des}}$ is as expected. Now we should quantitatively analyze the phase bias φ_{bias}. The phase bias is due to some non-ideal effects such as the accumulation of imperfect degeneracy, so the phase difference is not exactly 0 or π. Note that the φ_{bias} is fixed no matter whether the injection is from B or C, because the unitary condition should be subjected. From the theoretical T_{cons}, T_{des} in Supplementary Fig. 22b, we can get:

$$\frac{T_{\mathrm{cons}}}{T_{\mathrm{des}}} = \left| \frac{\cos\theta + \sin\theta\, e^{i\varphi_{\mathrm{bias}}}}{\cos\theta - \sin\theta\, e^{i\varphi_{\mathrm{bias}}}} \right|^2 \tag{44}$$

And substitute the measured magnitude:

$$\frac{T_{\mathrm{cons}}}{T_{\mathrm{des}}} = \frac{T_{\mathrm{C-C}} + T_{\mathrm{C-B}} + 2\sqrt{T_{\mathrm{C-C}} T_{\mathrm{C-B}}} \cos\varphi_{\mathrm{bias}}}{T_{\mathrm{B-B}} + T_{\mathrm{B-C}} - 2\sqrt{T_{\mathrm{B-B}} T_{\mathrm{B-C}}} \cos\varphi_{\mathrm{bias}}} \tag{45}$$

We can get:

$$\varphi_{\mathrm{bias}} = \arccos\left[\frac{T_{\mathrm{cons}}(T_{\mathrm{B-B}} + T_{\mathrm{B-C}}) - T_{\mathrm{des}}(T_{\mathrm{C-C}} + T_{\mathrm{C-B}})}{2T_{\mathrm{cons}}\sqrt{T_{\mathrm{B-B}} T_{\mathrm{B-C}}} + 2T_{\mathrm{des}}\sqrt{T_{\mathrm{C-C}} T_{\mathrm{C-B}}}}\right] \tag{46}$$

Thus we can get the measured phase bias φ_{bias}, it should be zero in the ideal case. Supplementary Fig. 22c-d shows the magnitude and phase information of SO(2) with θ=3π/8. Supplementary Fig. 22c illustrates the measured transmission (relevant to the magnitude of SO(2) elements). Supplementary Fig. 22d illustrates the measured transmission of constructive (T_{cons}) and destructive (T_{des}), and the calculated phase bias φ_{bias}. The maximum phase bias φ_{bias} is around 0.3π.

The other phase information we should detect is the basic STIRAP of braiding. In theory, there will be a geometric phase π acquired after experiencing STIRAP. Supplementary Fig. 11c,e

shows the interference method. For the constructive setup, the beam is split into two lateral STIRAP and combined. For the destructive setup, two split beams go through STIRAP and straight reference waveguide, respectively. Considering the phase difference between two beams in a constructive setup is exactly zero, here we do not need to calculate φ_{bias}, but can directly calculate the geometric phase (π in theory) through the law of cosine, according to the transmission of constructive and destructive interference. The measured transmission spectra of constructive and destructive interference are shown in the inset of Supplementary Fig. 11e. Around 0.8π phase was observed in broadband (π in theory), which is calculated through the cosine theorem based on the transmission spectra of constructive and destructive interference.

Supplementary Figures

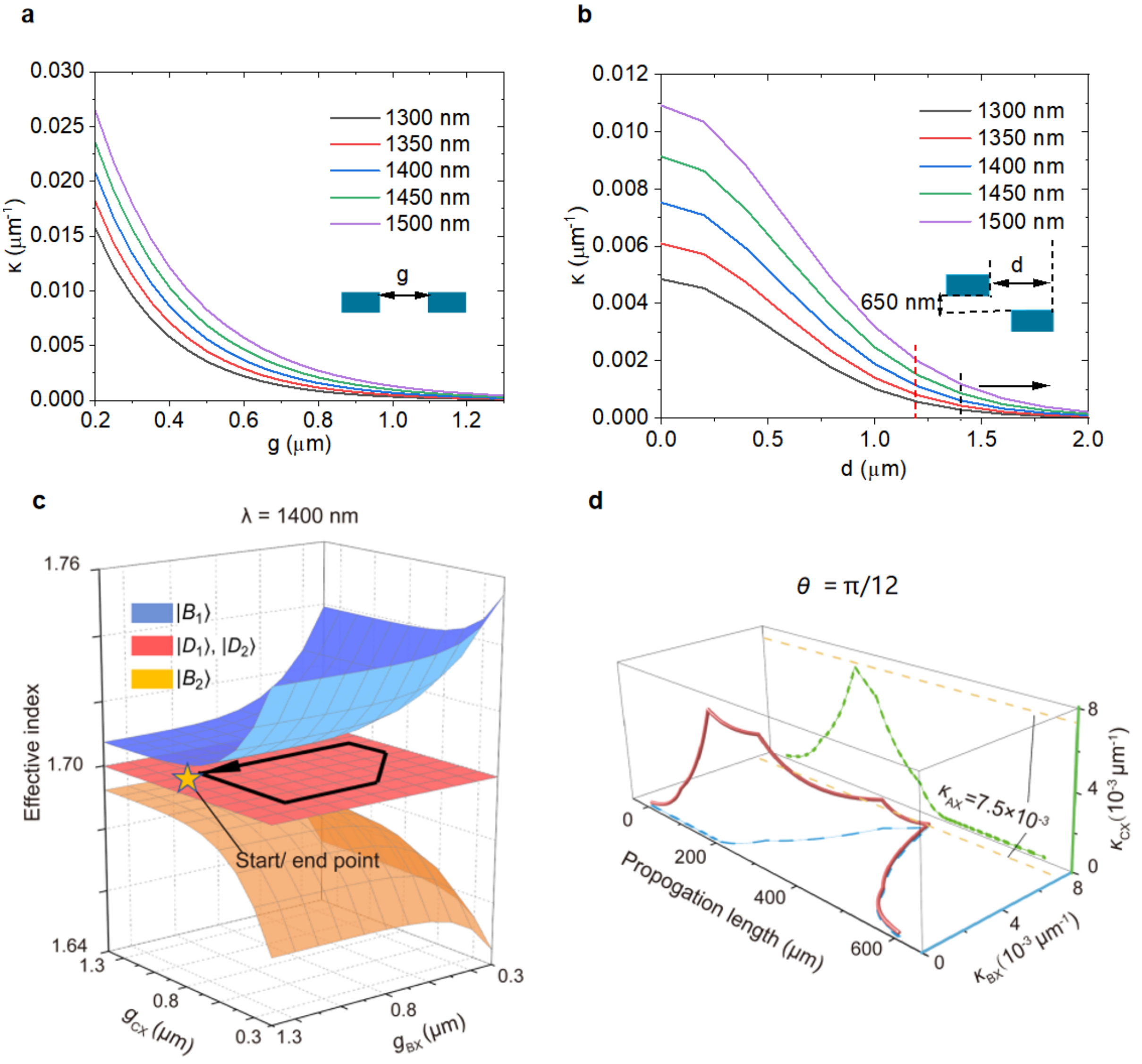

Supplementary Fig. 1| Coupling coefficient-distance relation and the holonomy accomplished through distance and coupling coefficients. a, Coupling coefficient between two waveguides (on the same layer) with different gaps at wavelength from 1300 nm to 1500 nm. κ is calculated through Lumerical Mode Solutions (see Methods). **b,** Coupling coefficients between two waveguides on different layers (taking inter-layer thickness to be 650 nm as an example) with different distances at wavelength from 1300 nm to 1500 nm. The designed holonomy path should ensure that unwanted coupling (κ_{AB}, κ_{AC}) is small enough, avoiding affecting the degeneracy. If the distance d is very small (e.g. smaller than the red dashed line position), the degeneracy will be affected remarkably. More degeneracy analyses are shown in Supplementary Note 2 and Supplementary Fig. 5. **c,** Eigenvalues of the four-site Hamiltonian as a function of g_{AX}, g_{BX}, where the black curve shows the holonomic path. Two eigenvectors ($|D_1\rangle$ and $|D_2\rangle$, see Supplementary Equation (15)) are degenerated, spanning the two-fold

degenerate subspace, and the holonomic evolution is accomplished by an adiabatic cyclic loop of g_{AX} and g_{BX}. **d,** Coupling coefficient evolution for SO(2) that $\theta=\pi/12$ (mathematically calculated).

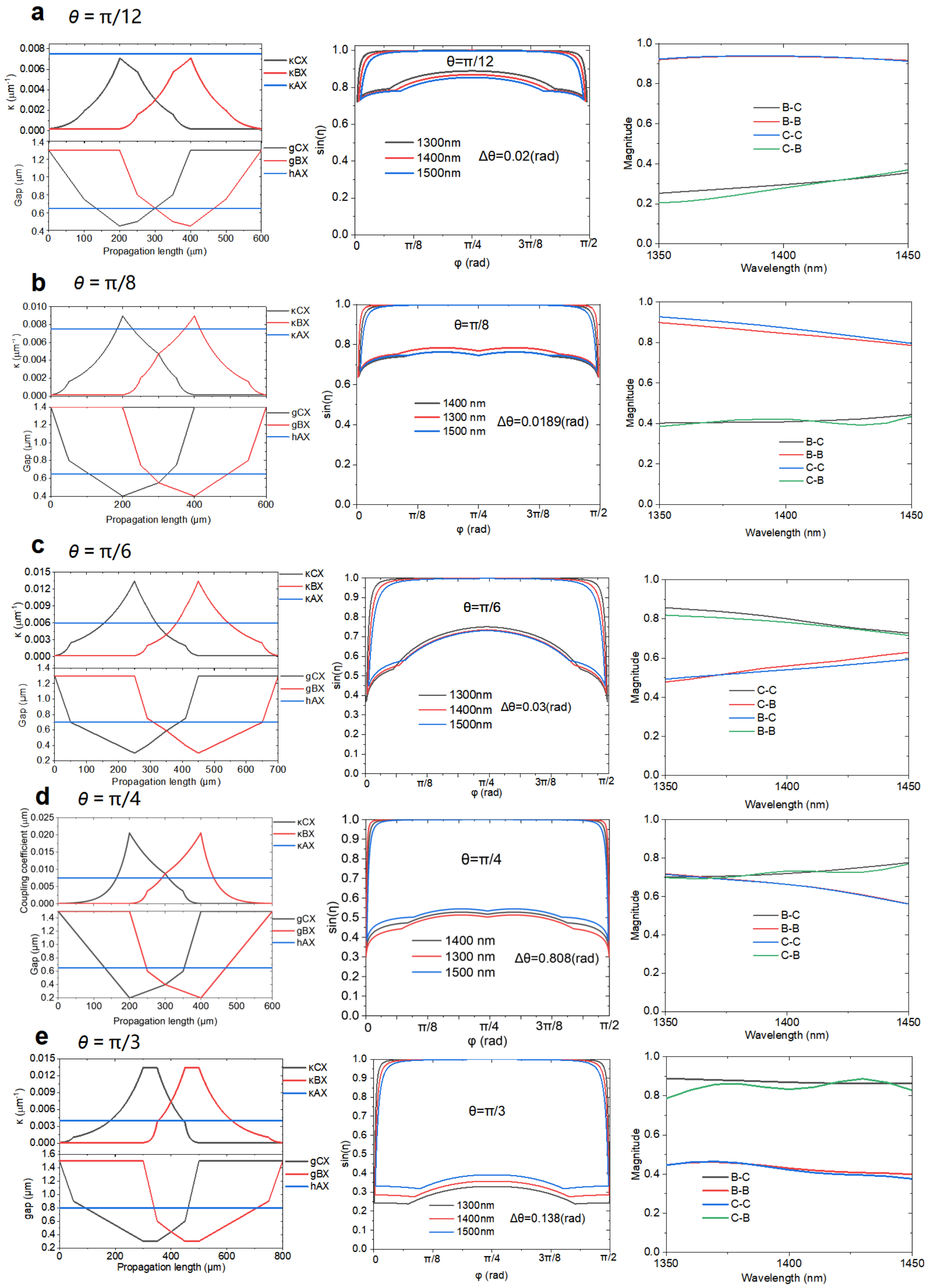

Supplementary Fig. 2| Parameters of unitary matrix simulated in Supplementary Fig. 31. Left column: g_{AX}, g_{BX}, g_{CX} and κ_{AX}, κ_{BX}, κ_{CX} in whole propagation. Central column: Mathematical integral of Wilczek-Zee connection at wavelength from 1300 nm to 1500 nm,

we can see the parameter path is relatively stable to wavelength. Right column: Output magnitude from 3D FDTD simulation. **a,** θ=π/12. **b,** θ=π/8. **c,** θ=π/6. **d,** θ=π/4. **e,** θ=π/3.

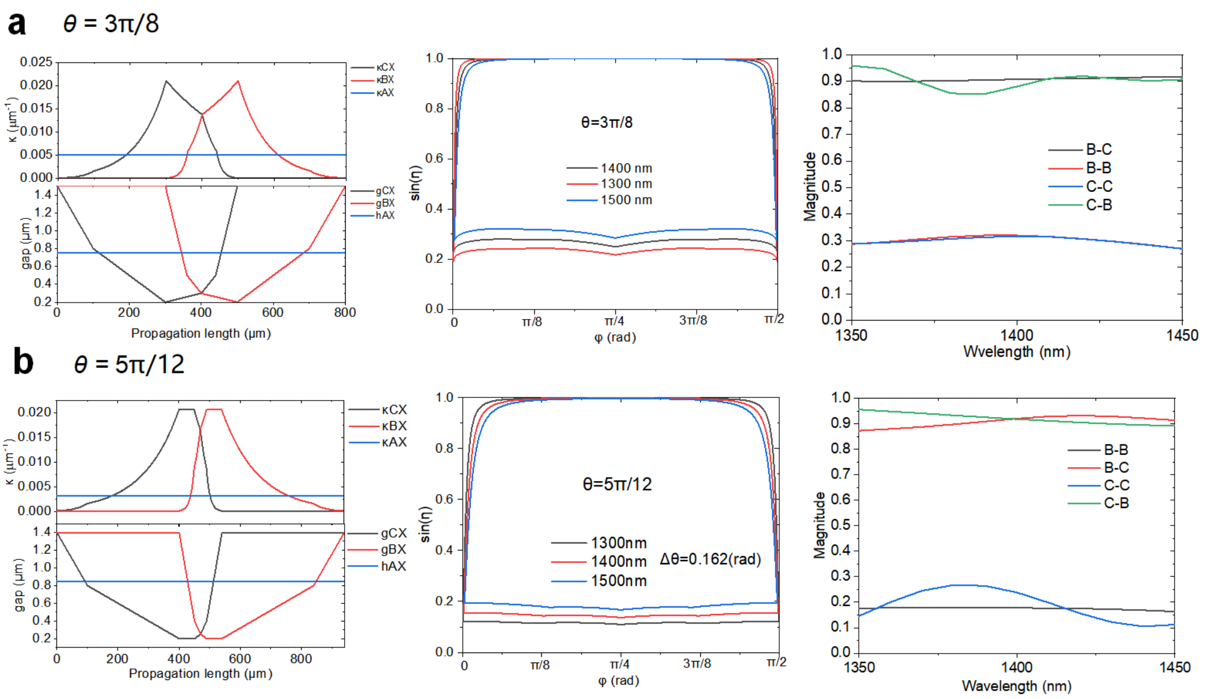

Supplementary Fig. 3| Parameters of unitary matrix simulated in Supplementary Fig. 31. Left column: g_{AX}, g_{BX}, g_{CX} and κ_{AX}, κ_{BX}, κ_{CX} in whole propagation. Central column: Mathematical integral of Wilczek-Zee connection at wavelength from 1300 nm to 1500 nm, we can see the parameter path is relatively stable to wavelength. Right column: Output magnitude from 3D FDTD simulation. **a,** θ=3π/8. **b,** θ=5π/12.

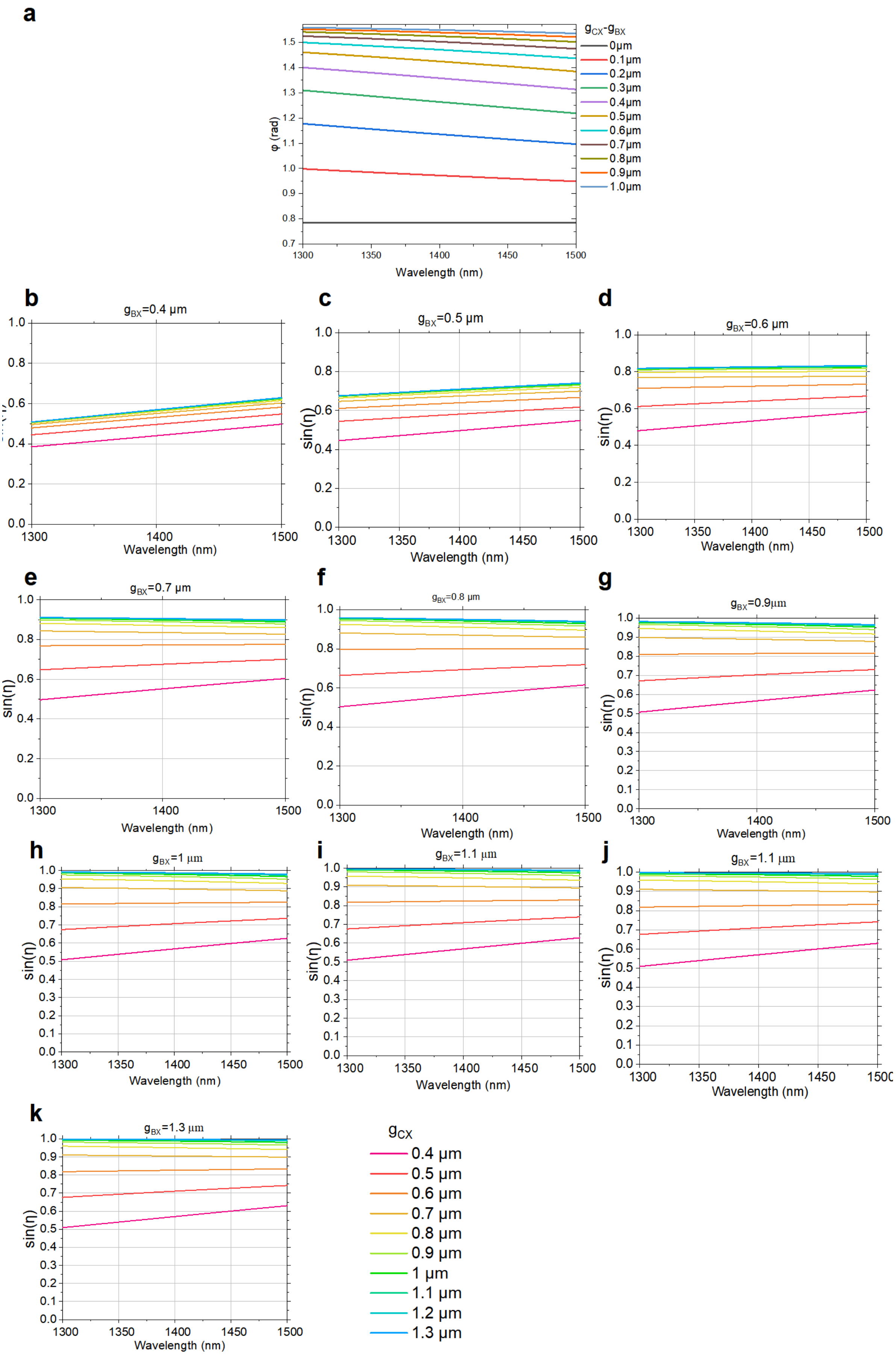
a
b
c
d
e
f
g
h
i
j
k
φ (rad)
Wavelength (nm)
g_{CX}-g_{BX}
0μm
0.1μm
0.2μm
0.3μm
0.4μm
0.5μm
0.6μm
0.7μm
0.8μm
0.9μm
1.0μm
g_{BX}=0.4 μm
g_{BX}=0.5 μm
g_{BX}=0.6 μm
g_{BX}=0.7 μm
g_{BX}=0.8 μm
g_{BX}=0.9μm
g_{BX}=1 μm
g_{BX}=1.1 μm
g_{BX}=1.1 μm
g_{BX}=1.3 μm
sin(η)
g_{CX}
0.4 μm
0.5 μm
0.6 μm
0.7 μm
0.8 μm
0.9 μm
1 μm
1.1 μm
1.2 μm
1.3 μm

Supplementary Fig. 4| φ, sin(η) at different wavelength. a, Calculated φ at wavelength from 1300 nm~1500 nm. **b-k,** Calculated sin(η) at wavelength from 1300 nm~ 1500 nm.

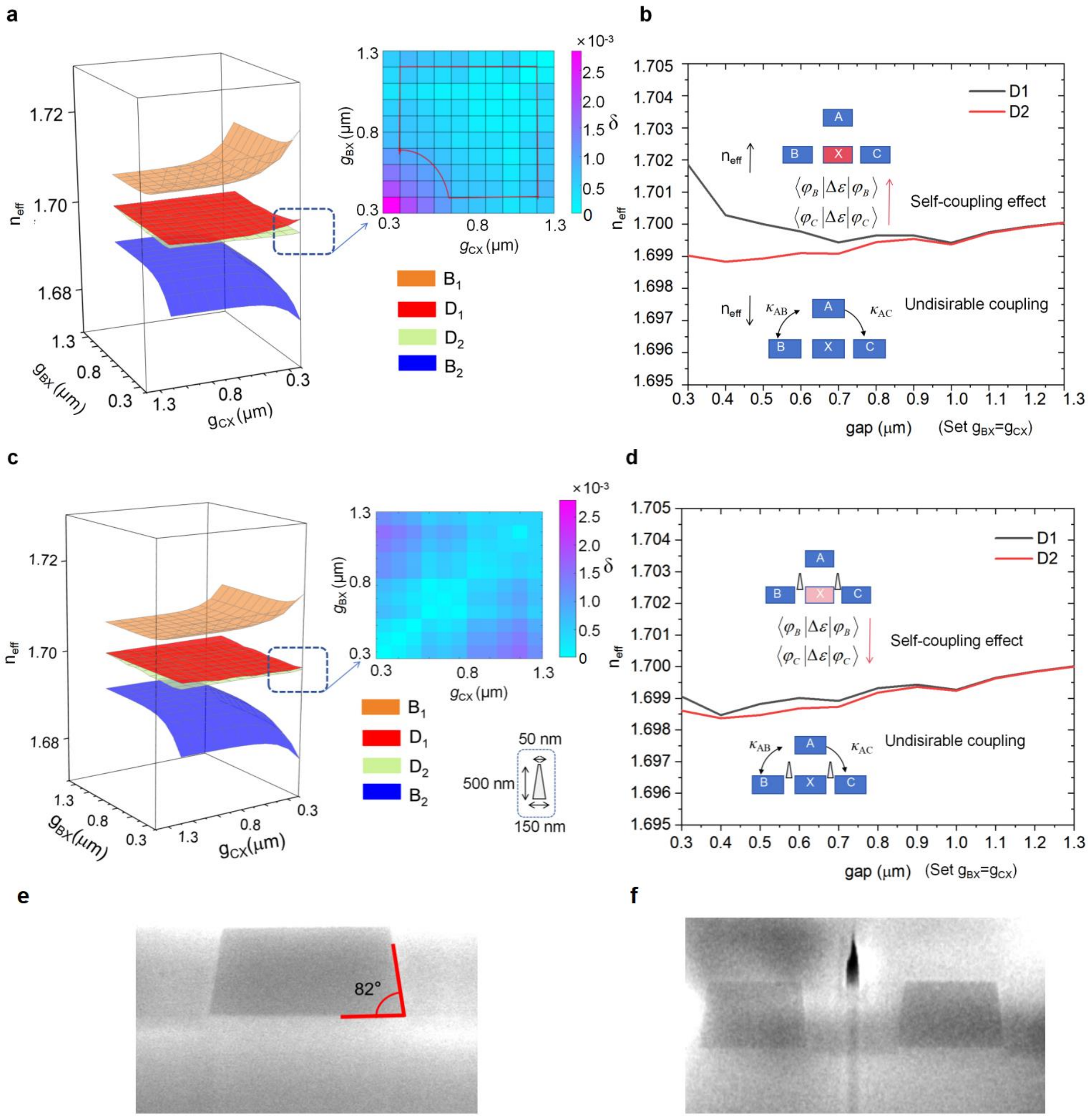

Supplementary Fig. 5| Non-perfect degeneracy in actual devices. a,c, Effective indices of four modes calculated through Lumerical Mode Solutions at wavelength 1400 nm as g_{BX} and g_{CX} change. The inset shows the difference δ between the effective indices of two degenerate modes (D_1, D_2). **a,** Without voids. **c,** With voids. **b,d,** Effective indices of two degenerate modes (D_1, D_2) as g_{BX} and g_{CX} change (set $g_{\mathrm{BX}} = g_{\mathrm{CX}}$). The insets show the undesirable effects. **b,** Without voids. **d,** With voids. **e,** The FIB image of trapezoidal. **f,** The FIB image of void in two waveguides.

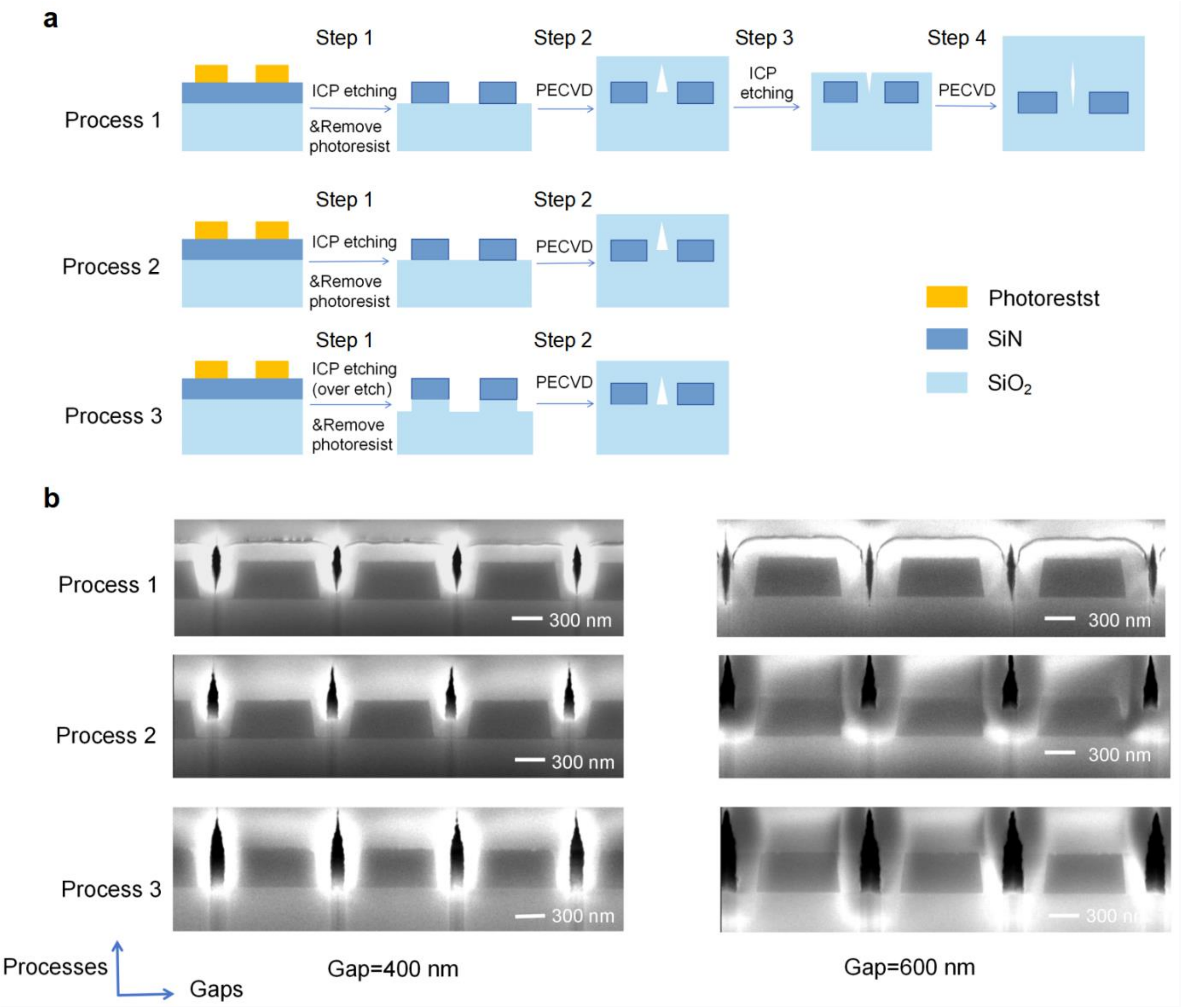

Supplementary Fig. 6| Different voids generated through different processes. a, Different processes can generate different voids. Process 2 is an ordinary operation, after the photoresist is patterned, ICP etching is utilized in Step 1 to etch silicon nitride. Then after removing the photoresist, silica is deposited in Step 2 through PECVD. Different from process 2, process 3 is over-etching around 200 nm in Step 1. Thus, the voids generated during PECVD in Step 2 have a lower position and larger size. Process 1 is based on process 2. After depositing 650 nm silica through PECVD in Step 2, ICP etching is utilized again in Step 3 to etch silica around 400 nm. Thus, the closure of voids reopens and becomes an inverted triangle shape. Finally, PECVD is utilized again to deposit silica in Step 4, and we can obtain smaller voids. **b,** FIB images of voids generated through different processes and different gaps. Each image represents the same process and gap, in which the array of voids have similar appearances, illustrating the reproducibility.

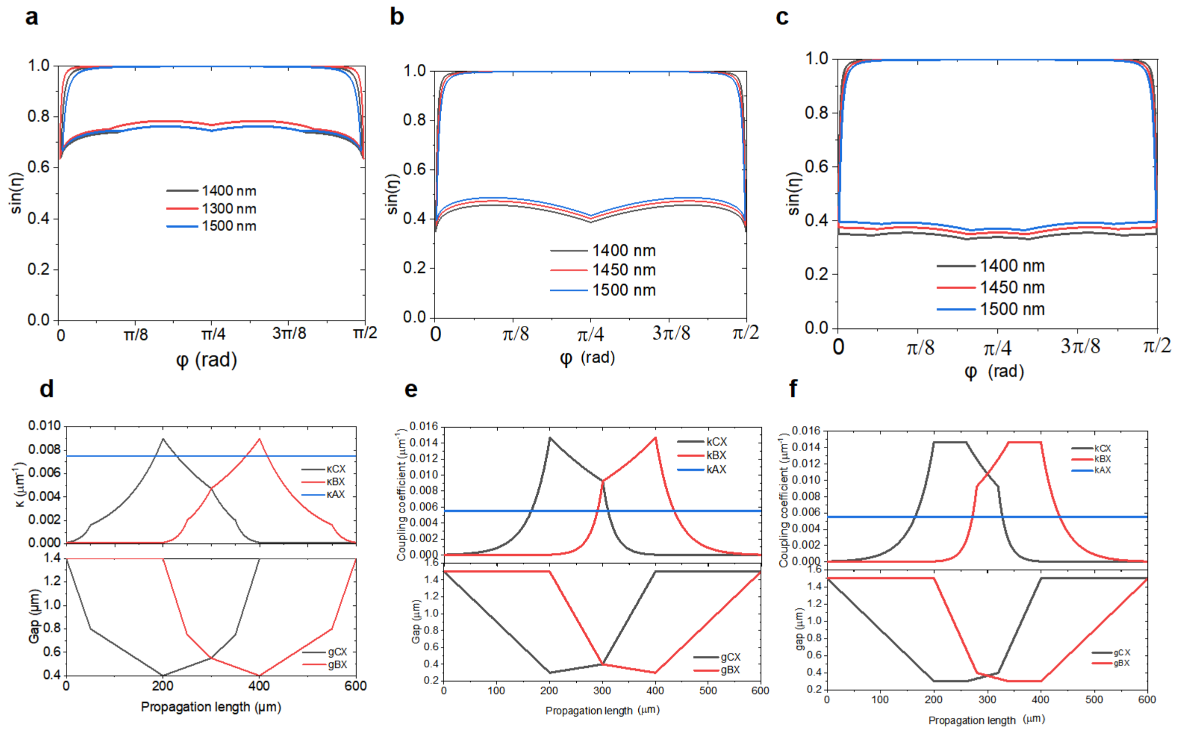

Supplementary Fig. 7| Unitary matrices in Fig. 2 in the main text. a-c, Mathematical integral of Wilczek-Zee connection at wavelength from 1300 nm to 1500 nm, we can see the parameter path is relatively stable to wavelength. **a,** θ=π/8. **b,** θ=5π/18. **c,** θ=π/3. **d-f,** The gap and coupling coefficients: g_{AX}, g_{BX}, g_{CX} in whole propagation, κ_{AX}, κ_{BX}, κ_{CX} in whole propagation. **d,** θ=π/8. **e,** θ=5π/18. **f,** θ=π/3.

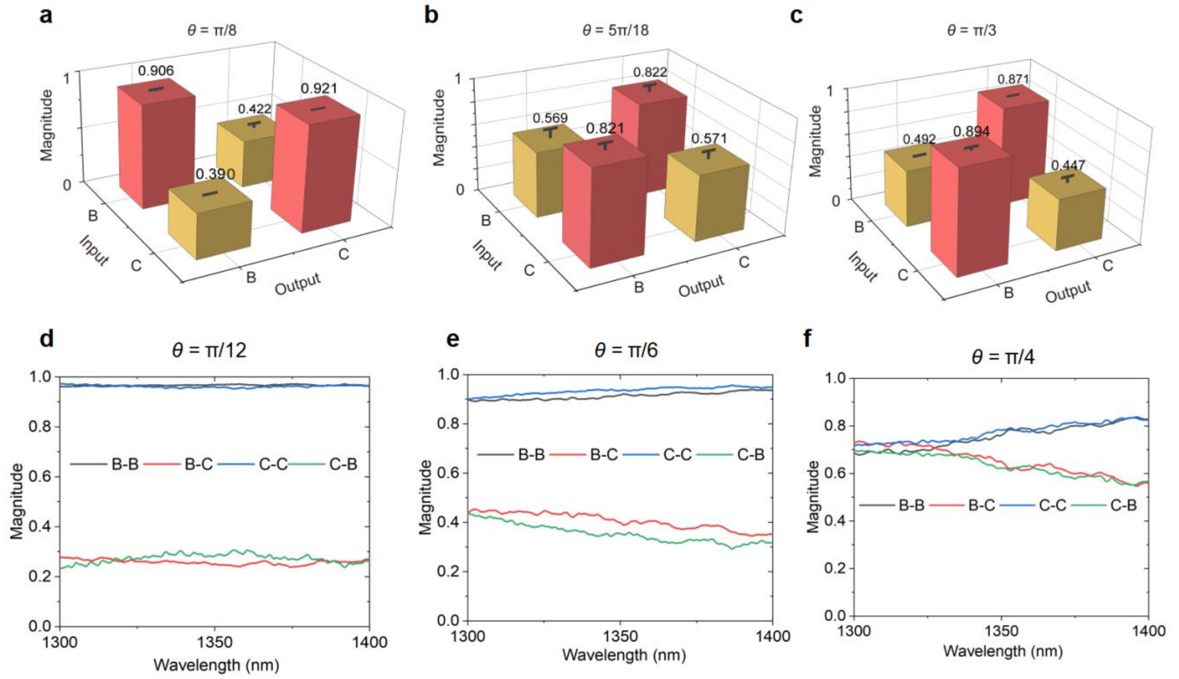

Supplementary Fig. 8| The experimental results for different SO(2). a-c, Measured elements of three typical SO(2) with $e^{i\theta\sigma_y}$ at wavelength 1450 nm after normalization, whose transmission spectra are in the main manuscript Fig. 2a-c, i.e., **a,** θ=π/8. **b,** θ=5π/18. **c,** θ=π/3. **d-f,** Magnitude-wavelength of some other elements of SO(2). **d,** θ=π/12. **e,** θ=π/6. **f,** θ=π/4.

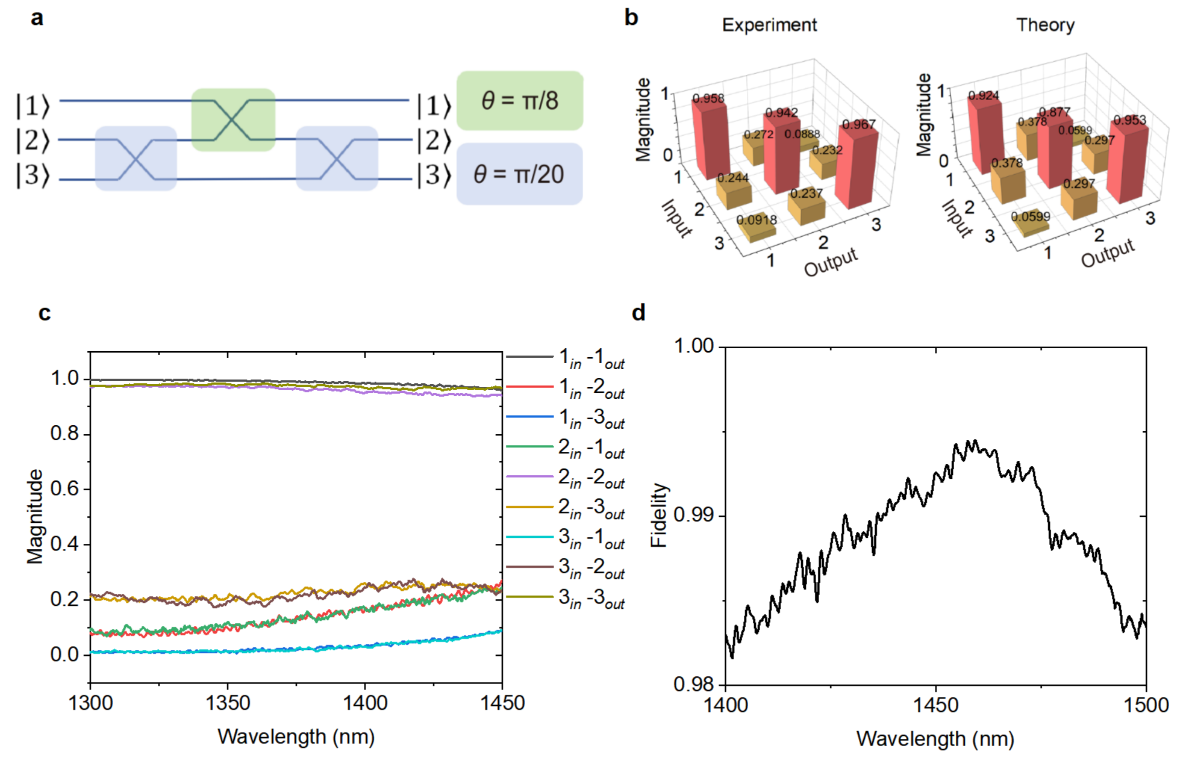

Supplementary Fig. 9| Broadband characteristic of an element of SO(3) and metasurface information. a-c, Broadband characteristic of a SO(3). **a,** Rotation information of a SO(3). **b,** The measured elements of the SO(3) at 1450 nm and theoretically predicted elements. **c,** Measured transmission of the SO(3). **d,** Fidelity-wavelength relation of the SO(3).

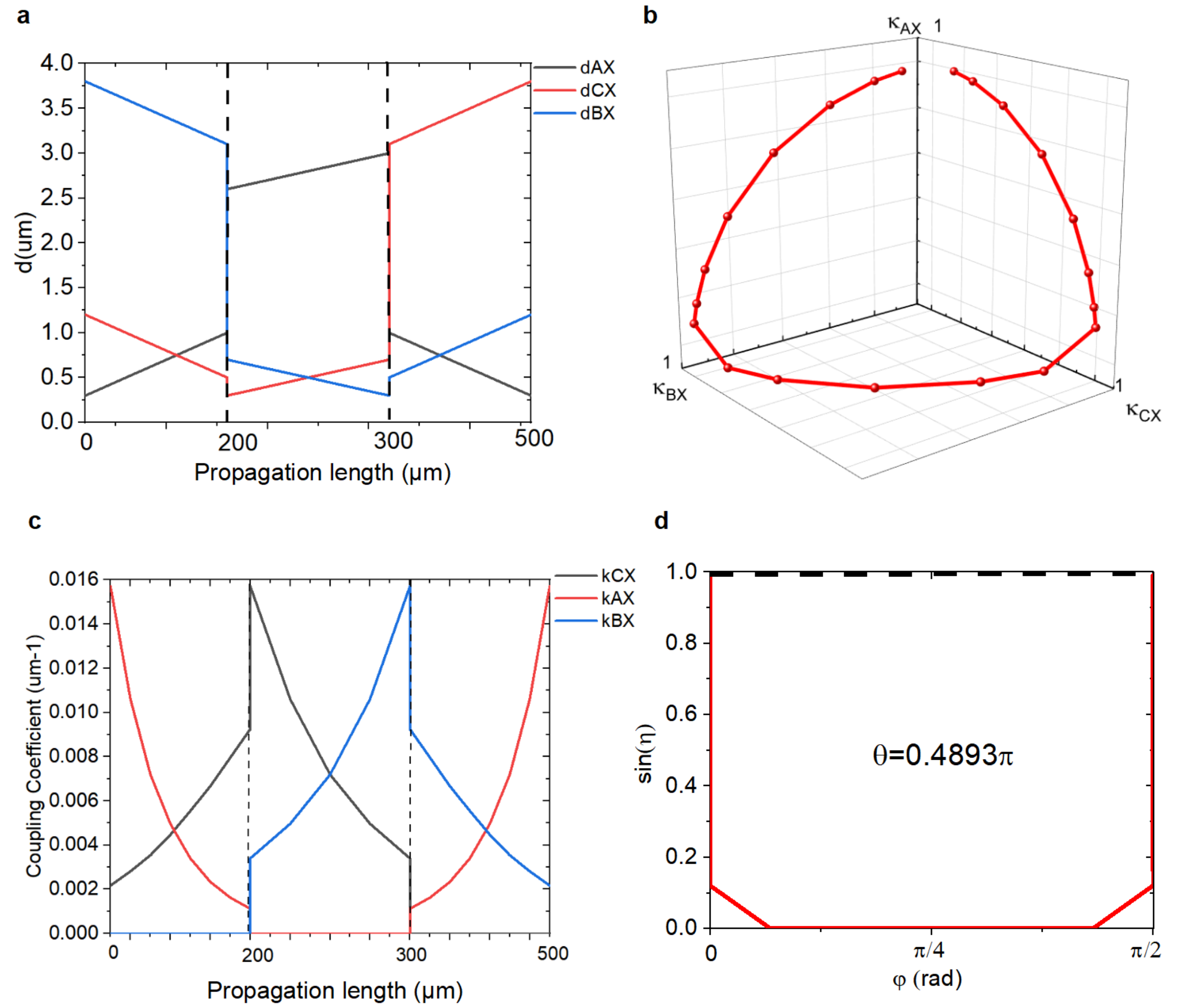

Supplementary Fig. 10| Gap and coupling coefficient evolution in two-mode braiding. a, g_{AX}, g_{BX}, g_{CX} in three steps. **b,** Normalize κ_{AX}, κ_{BX}, κ_{CX} to a 2-sphere through $\boldsymbol{\kappa}/|\boldsymbol{\kappa}|$. As we can see, although coupling coefficients are discontinuous due to waveguide X, it is continuous in $\boldsymbol{\kappa}$ space after normalization, which means the eigenmodes are continuous at discontinuous points. **c,** κ_{AX}, κ_{BX}, κ_{CX} in three steps. **d,** Mathematical integral of Wilczek-Zee connection.

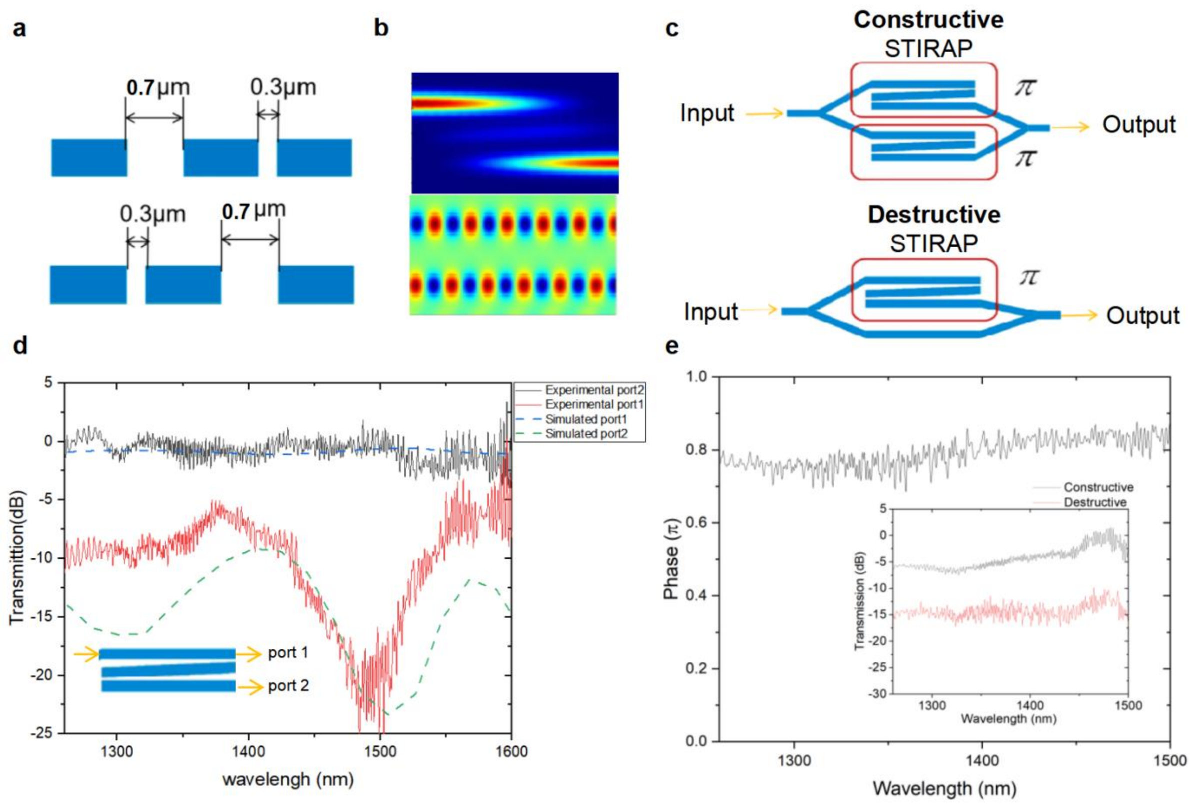

Supplementary Fig. 11| Lateral stimulated Raman adiabatic passage (STIRAP) a, The cross-section of lateral STIRAP at initial and final positions, and their detailed parameters, the length is around 200 μm. **b,** Simulated power transfer and geometric phase π acquired, compared with a reference isolated waveguide at the output position. **c,** Schematic of the measurement of the geometric phase through interference. For the constructive setup, the beam is split into two lateral STIRAP and combined together. For the destructive setup, two split beams enter STIRAP and a straight reference waveguide, respectively. **d,** Experimental and simulated results for power transfer. **e,** Experimental observation of geometric phase. Around 0.8π phase was observed, which is calculated through the cosine theorem based on the transmission of constructive and destructive interference.

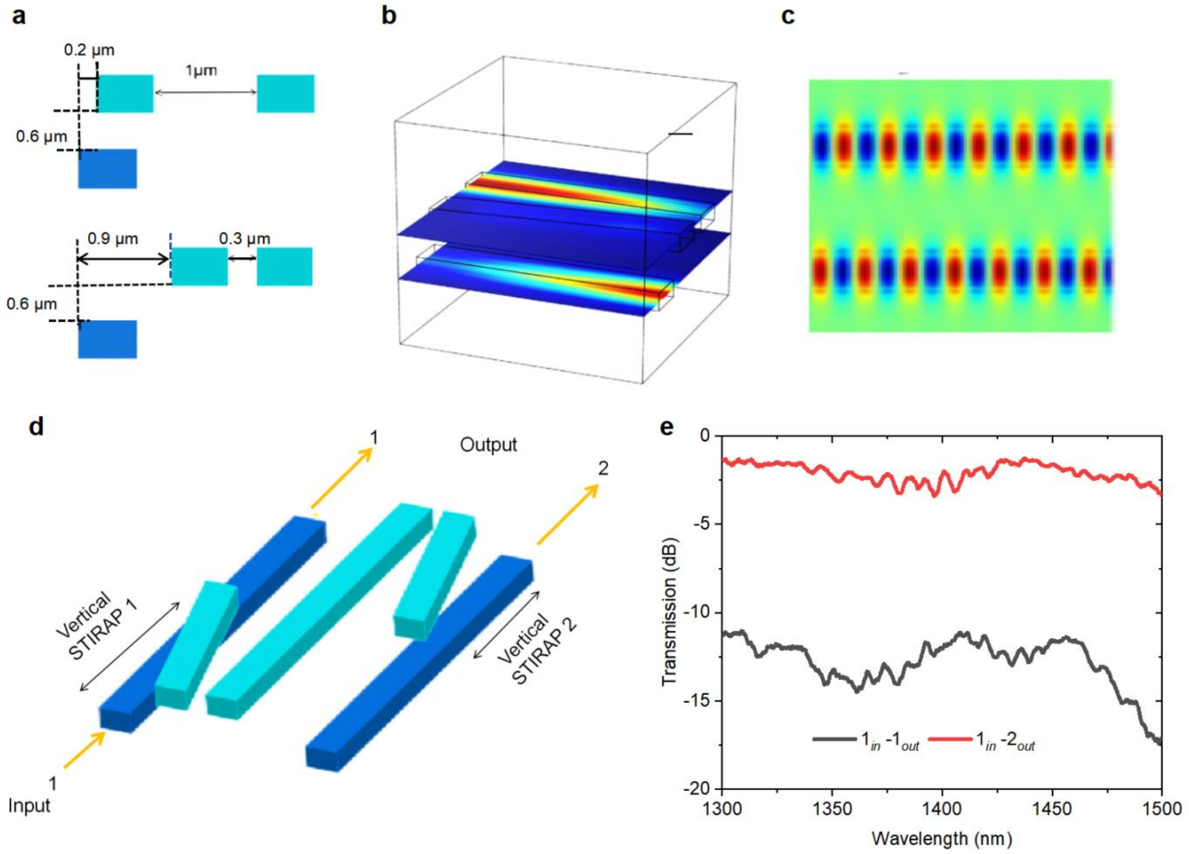

Supplementary Fig. 12| Vertical stimulated Raman adiabatic passage (STIRAP). a, The cross-section of vertical STIRAP at initial and final positions, and their detailed parameters, the length is around 200 μm. **b,** Simulated power transfer at wavelength 1500 nm through COMSOL. **c,** Geometric phase π acquired, compared with a reference isolated waveguide at the output position. **d,** Experimental structure for power transfer after two vertical STIRAP. **e,** Experimental result for power transfer after two vertical STIRAP.

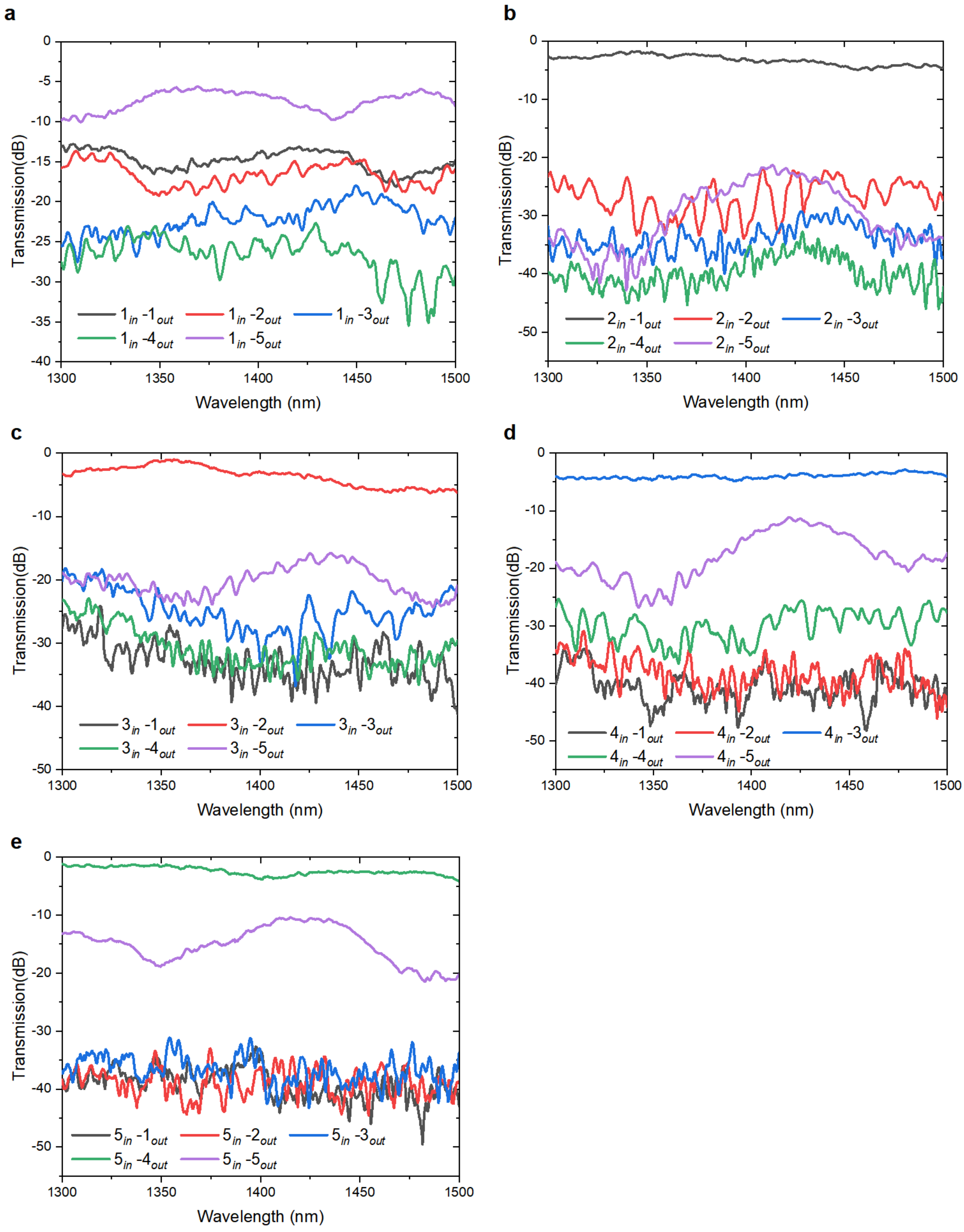

Supplementary Fig. 13| Transmission spectra of five-mode braiding in the main text. a, Measured transmission spectra input from port 1. **b,** Measured transmission spectra input from port 2. **c,** Measured transmission spectra input from port 3. **d,** Measured transmission spectra input from port 4. **e,** Measured transmission spectra input from port 5.

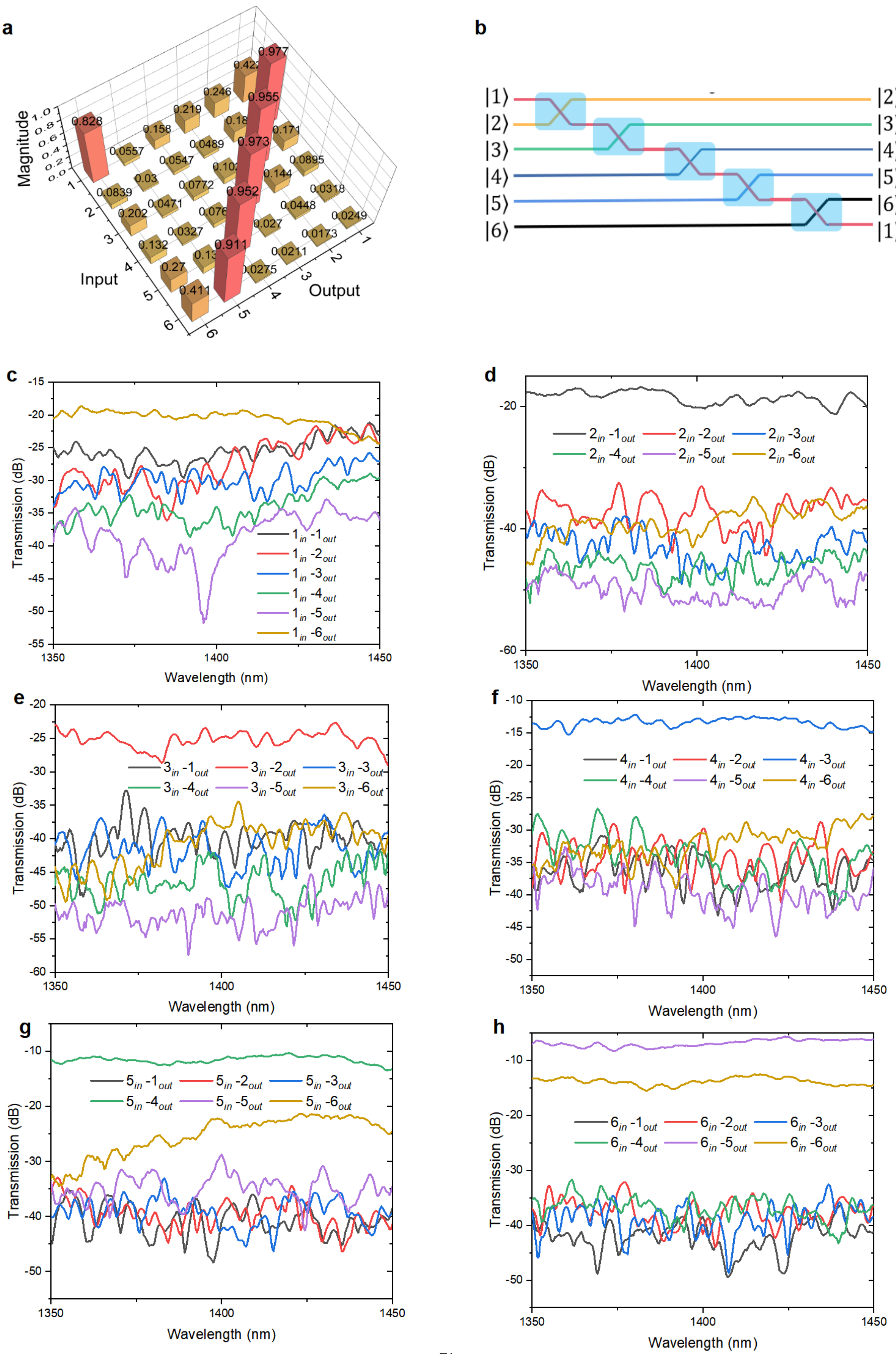
a
Magnitude
Input
Output
0.828
0.977
0.955
0.973
0.952
0.911
0.411
0.0557
0.158
0.219
0.246
0.171
0.0895
0.0318
0.0249
0.0173
0.0211
0.0275
0.0547
0.0489
0.144
0.0448
0.027
0.0839
0.202
0.132
0.27
0.03
0.0471
0.0327
0.0772
b
|1⟩
|2⟩
|3⟩
|4⟩
|5⟩
|6⟩
|2⟩
|3⟩
|4⟩
|5⟩
|6⟩
|1⟩
c
Transmission (dB)
Wavelength (nm)
1in -1out
1in -2out
1in -3out
1in -4out
1in -5out
1in -6out
d
Transmission (dB)
Wavelength (nm)
2in -1out
2in -2out
2in -3out
2in -4out
2in -5out
2in -6out
e
Transmission (dB)
Wavelength (nm)
3in -1out
3in -2out
3in -3out
3in -4out
3in -5out
3in -6out
f
Transmission (dB)
Wavelength (nm)
4in -1out
4in -2out
4in -3out
4in -4out
4in -5out
4in -6out
g
Transmission (dB)
Wavelength (nm)
5in -1out
5in -2out
5in -3out
5in -4out
5in -5out
5in -6out
h
Transmission (dB)
Wavelength (nm)
6in -1out
6in -2out
6in -3out
6in -4out
6in -5out
6in -6out
1350
1400
1450

Supplementary Fig. 14| Experimental results of six-mode braiding. a, Measured output magnitude of six-mode non-Abelian braiding with different inputs at wavelength 1400 nm after normalization. **b,** Simplified mathematical model. **c-h,** Transmission spectra with different inputs from ports 1 to 6.

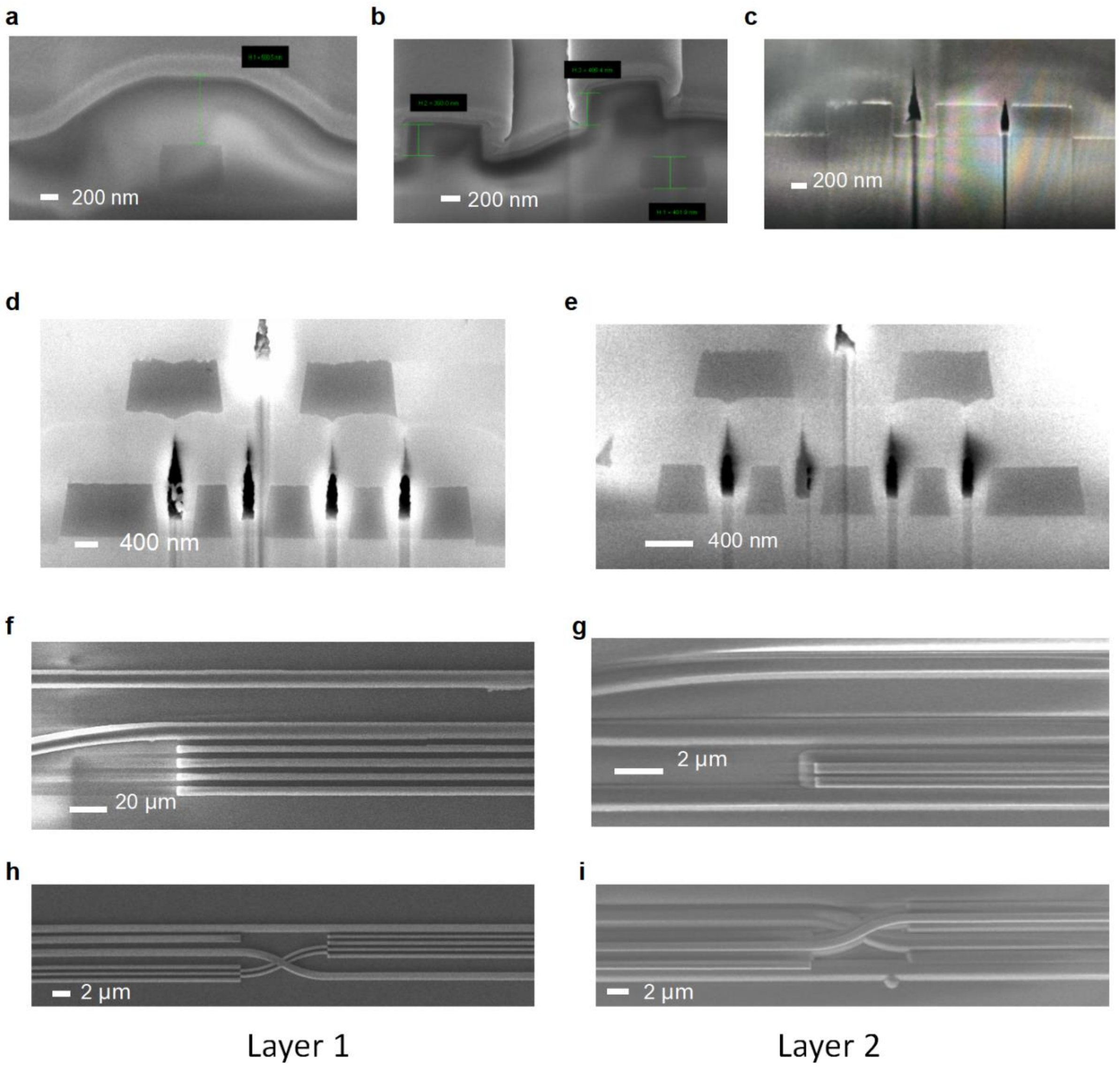

Supplementary Fig. 15| Planarization for two-layer silicon nitride. a, Punches on the silica surface after PECVD silica on SiN waveguides**. b,** Twisted and deformed SiN waveguides on layer 2 without planarization. **c,** Voids generated by deficient filling gap capability of PECVD, early closure point can be observed. **d,e,** Relatively flat surface for layer 2. **f,h,** SEM graphs of main waveguides and supporting waveguides on layer 1. **g,i,** SEM graphs of main waveguides on layer 2.

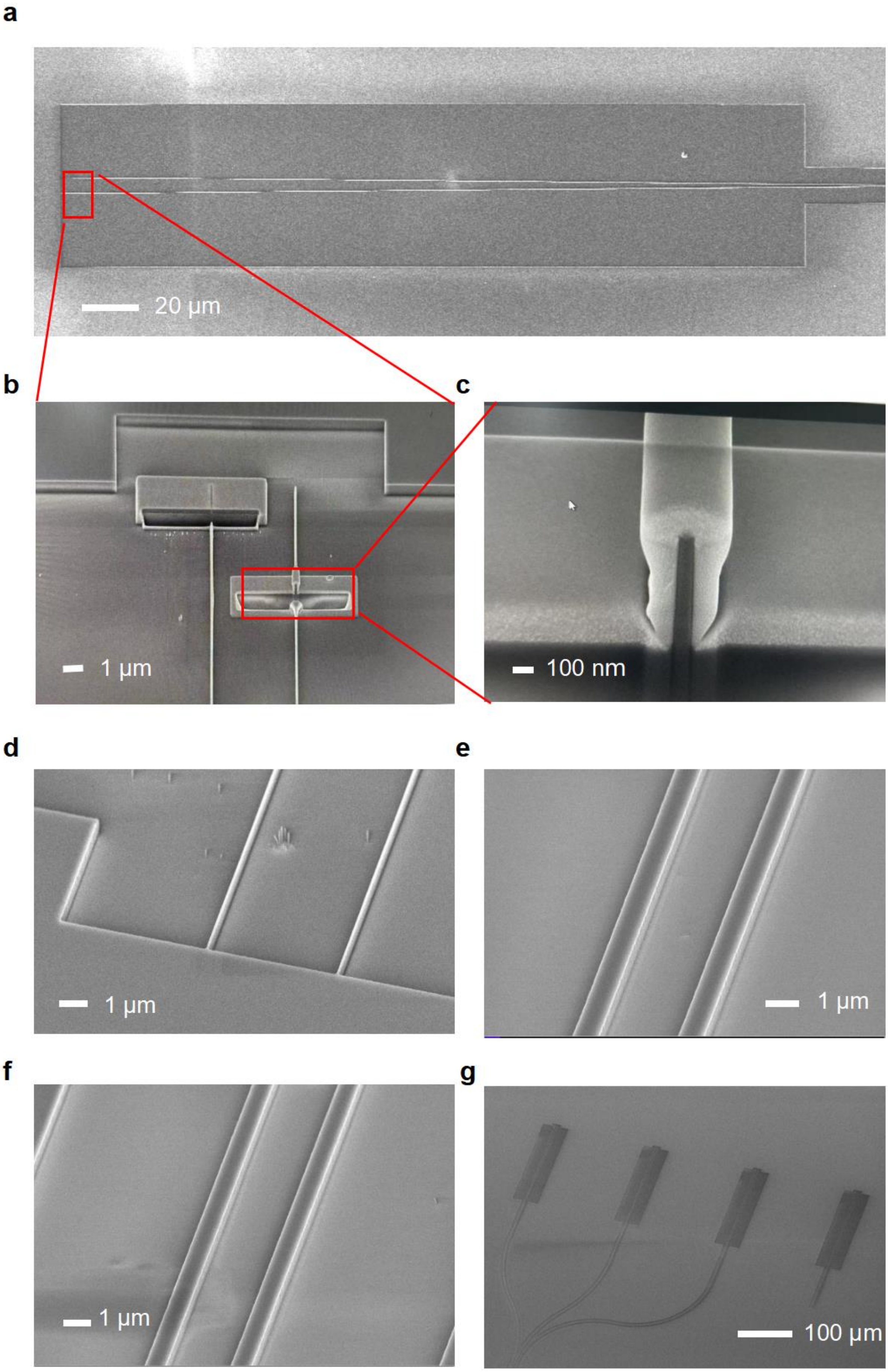
a
20 μm
b
1 μm
c
100 nm
d
1 μm
e
1 μm
f
1 μm
g
100 μm

Supplementary Fig. 16| Fork edge coupler. On the left side of the edge coupler, the width of the tips is 150 nm, and the center-center gap between the two tips is 4.55 μm. The whole length is about 400 μm for enough adiabatic evolution. **a,** The top-view SEM figure of the edge coupler. **b,** The SEM figure of the tips. **c,** The cross-section of the tips. **d-f,** SEM figures of the tips. **g,** The SEM figure of the edge coupler array.

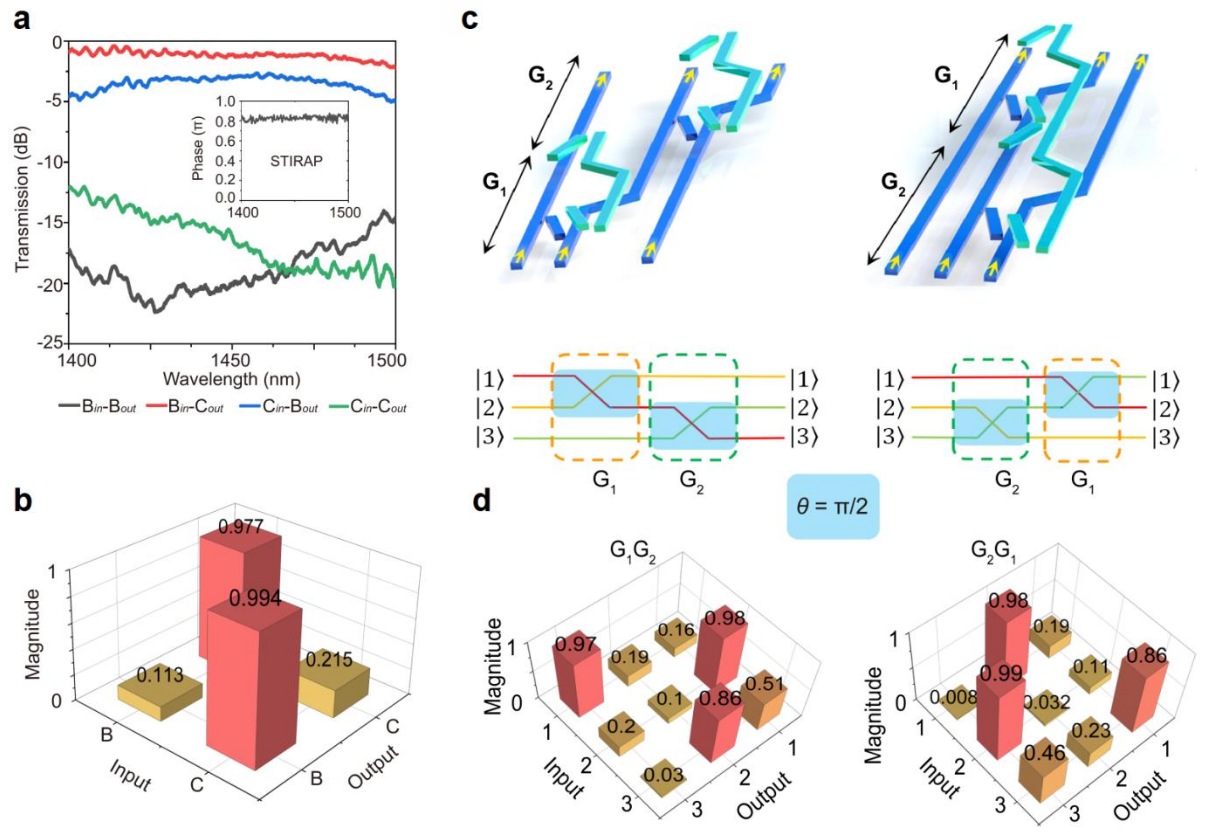

Supplementary Fig. 17| Structure and experimental results of two-mode braiding and three-mode braidings. a,b, Experimental results for two-mode braiding. **a,** Experimental transmission spectra for two-mode braiding. The inset shows the measured geometric phase acquired after experiencing a lateral STIRAP through the interference method, around 0.8π phase was observed in broadband (π in theory). **b,** Measured output optical magnitude with different inputs at wavelength 1450 nm after normalization. **c,d,** Structure and experimental results of three-mode braidings, illustrating the non-Abelian characteristic. **c,** Structure diagram of two kinds of three-mode non-Abelian braidings (G_1G_2 and G_2G_1) and corresponding simplified models. **d,** Measured outputs of three-order non-Abelian braiding (G_1G_2 and G_2G_1) with different inputs at wavelength 1400 nm after normalization.

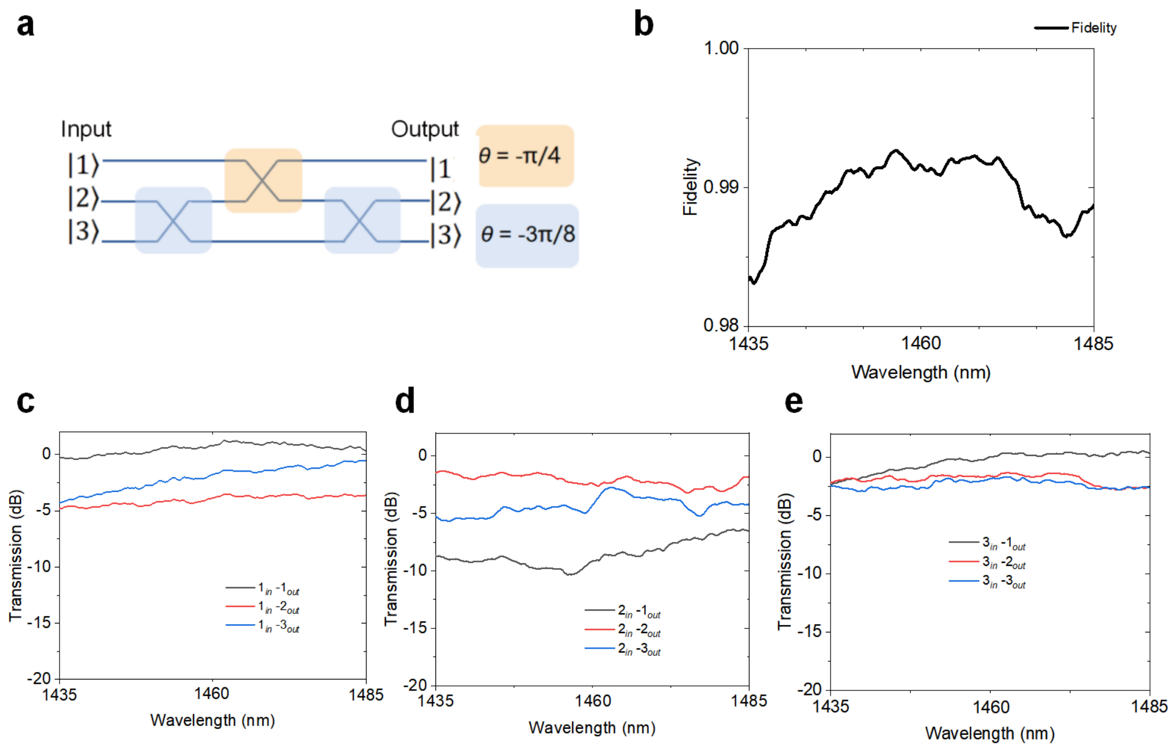

Supplementary Fig. 18| Mathematical model, fidelity-wavelength relation, and measured transmission spectra of SO(3) in the main text Fig. 2d. a, Rotation angles information. **b,** Fidelity-wavelength relation. **c-e,** Measured transmission spectra.

a

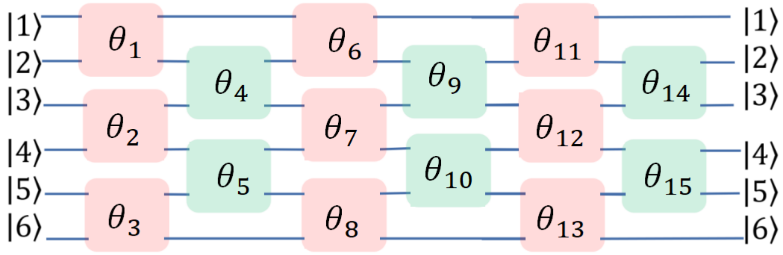

	θ_1	θ_2	θ_3	θ_4	θ_5	θ_6	θ_7	θ_8
(rad)	0.98	1.57	1.14	0.99	1.04	-0.72	1.32	-0.92

	θ_9	θ_{10}	θ_{11}	θ_{12}	θ_{13}	θ_{14}	θ_{15}	
(rad)	1.33	0.87	-0.82	0.81	1.18	0.64	0.63	

b

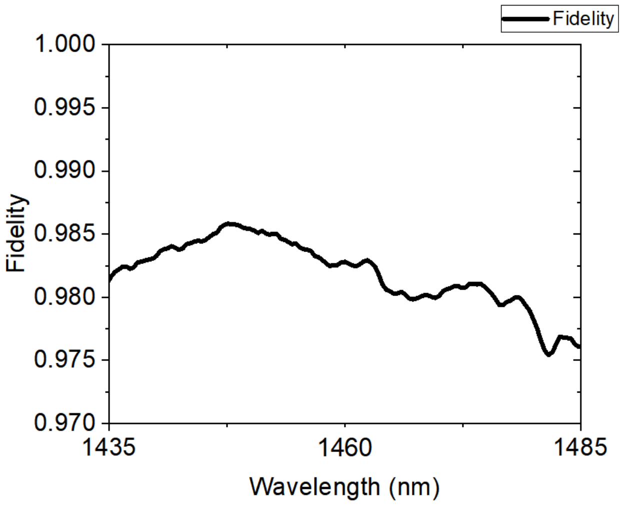

Supplementary Fig. 19| Experimental results for SO(6). a, The simplified model of the SO(6), the rotation angle is kept to two decimal places in radians. **b,** Fidelity-wavelength relation.

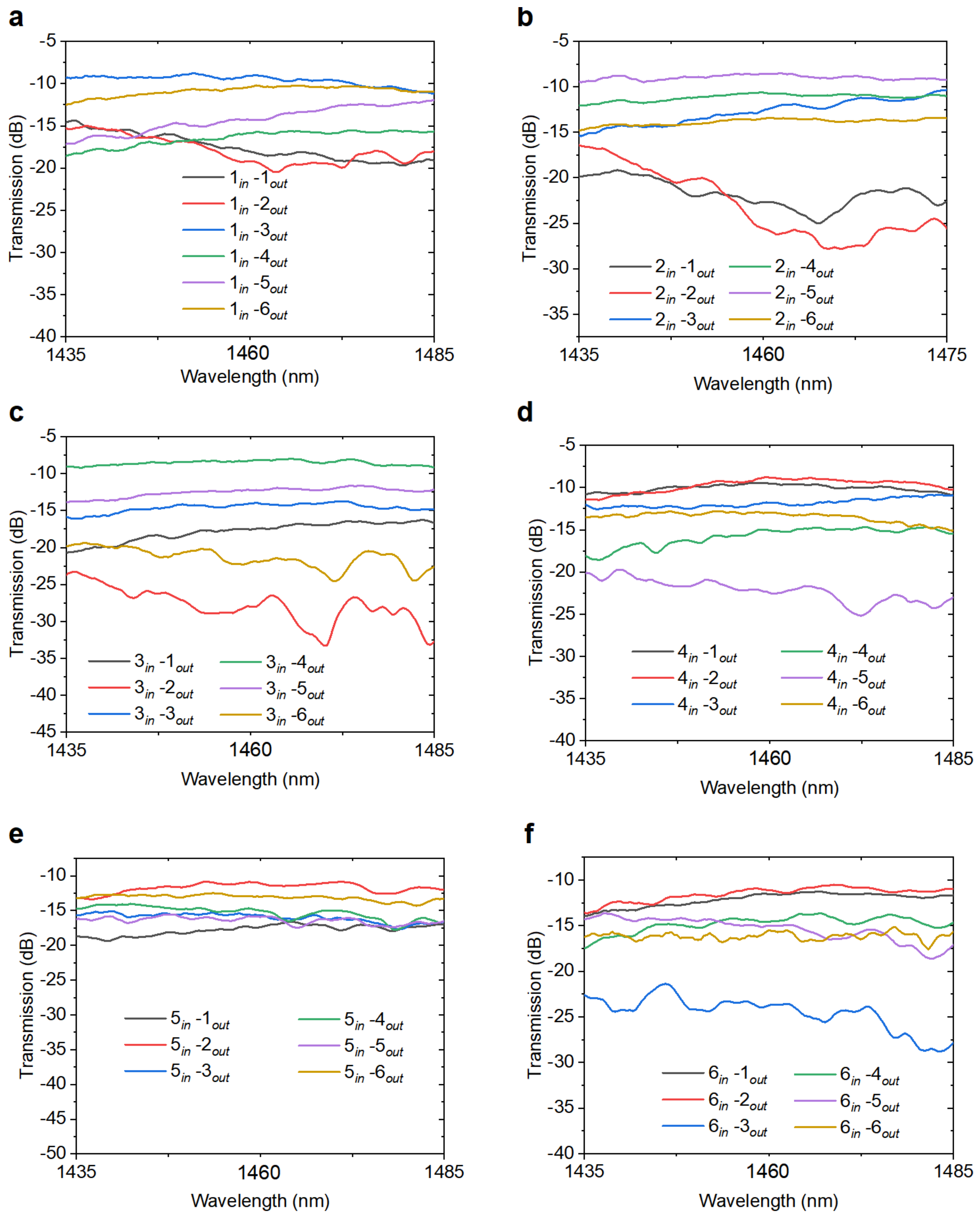

Supplementary Fig. 20| Measured transmission spectra for SO(6). a, Measured transmission spectra input from port 1. **b,** Measured transmission spectra input from port 2. **c,** Measured transmission spectra input from port 3. **d,** Measured transmission spectra input from port 4. **e,** Measured transmission spectra input from port 5. **f,** Measured transmission spectra input from the port 6.

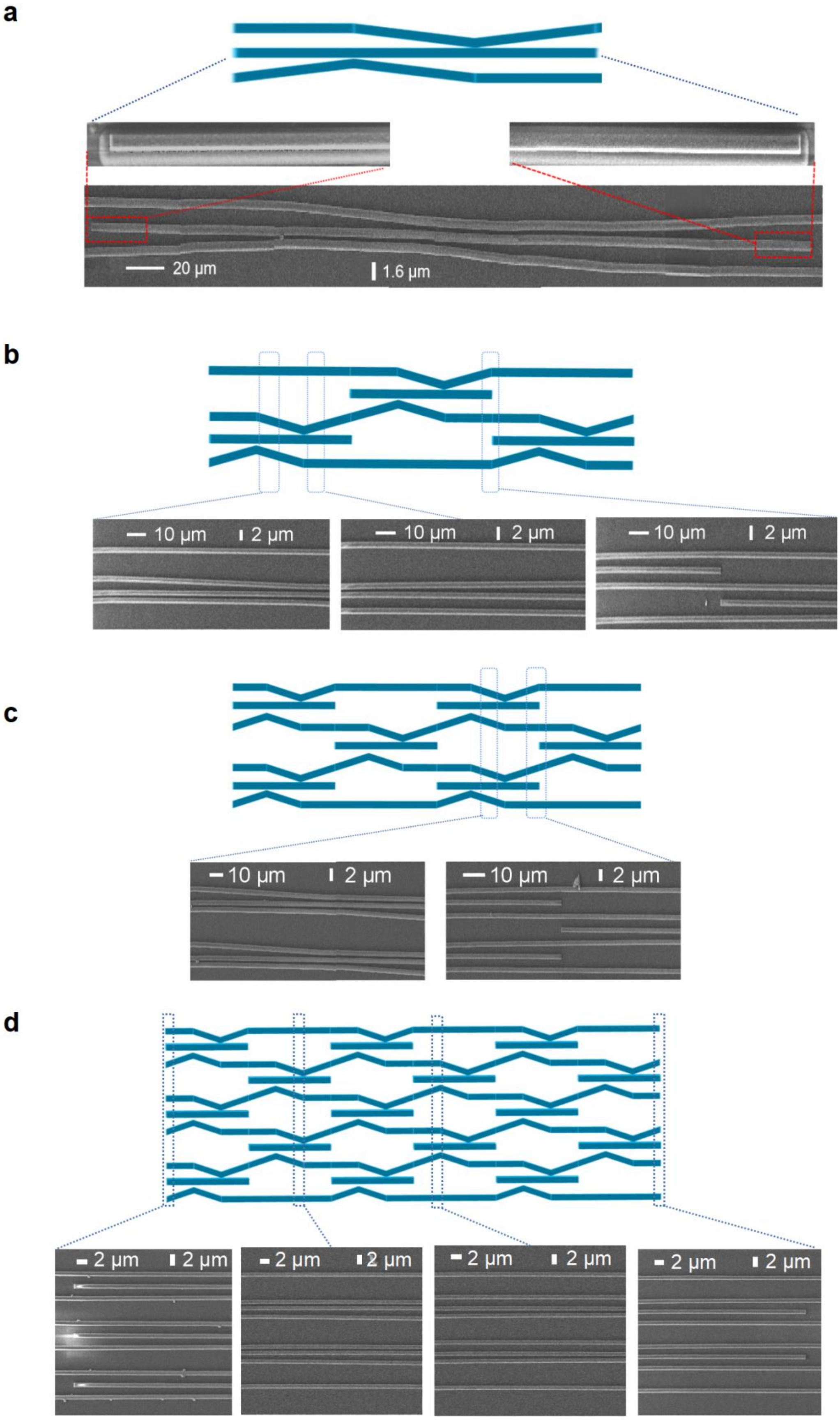
a
20 µm
1.6 µm
b
10 µm
2 µm
10 µm
2 µm
10 µm
2 µm
c
10 µm
2 µm
10 µm
2 µm
d
2 µm
2 µm
2 µm
2 µm
2 µm
2 µm
2 µm
2 µm

Supplementary Fig. 21| SEM figures for different devices at different positions. a, SO(2). The figures are scanning electron microscope (SEM) graphs of layer 1 and layer 2, through splicing and a combination of a series of SEM graphs. **b,** SEM graphs for layer 1 of SO(3) at different positions. **c,** SEM graphs for layer 1 of SO(4) at different positions. **d,** SEM graphs for layer 1 of SO(6) at different positions.

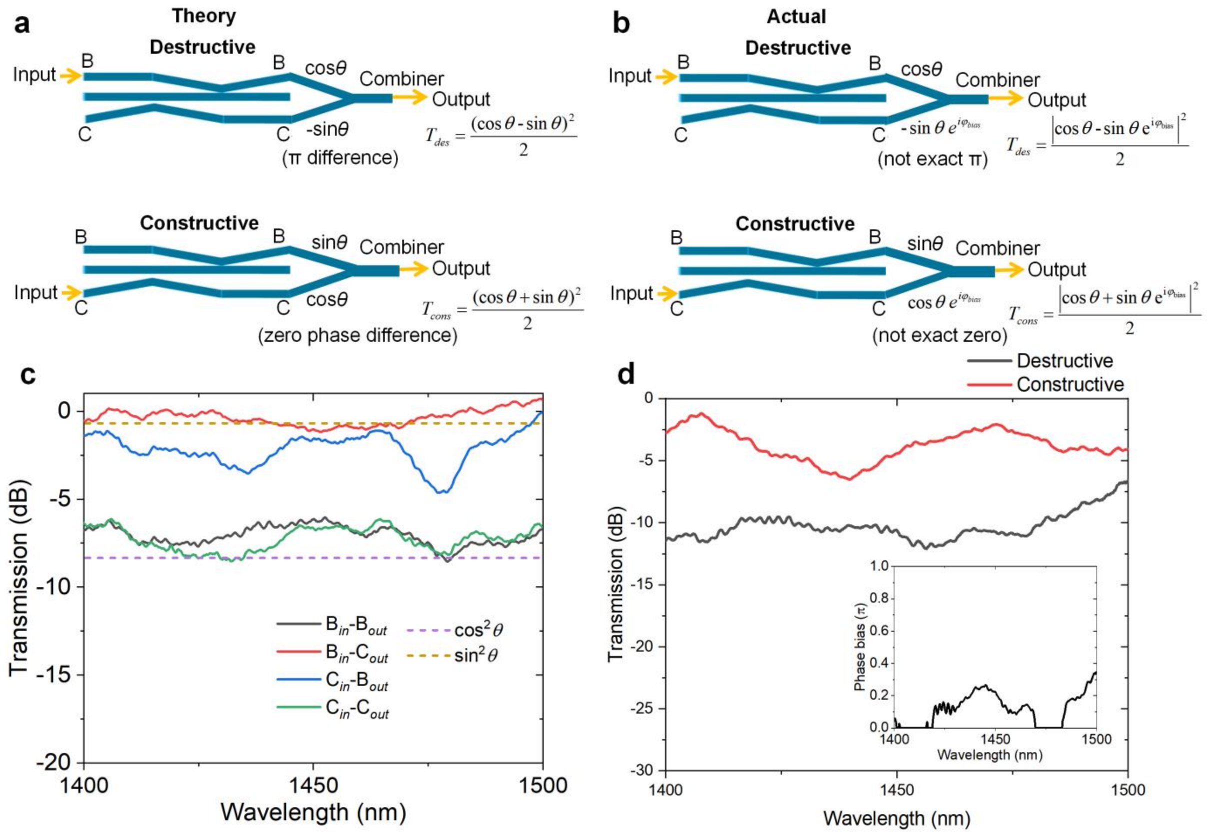

Supplementary Fig. 22| Interference method for SO(2). a, In theory, the phase difference between two beams is exactly 0 or π. **b,** In actual devices, there is a phase bias φ_{bias}, the phase difference between two beams is not exactly 0 or π. **c-d,** The magnitude and phase information of SO(2) with θ=3π/8. **c,** The measured transmission of SO(2) with θ=3π/8. Solid lines are experimental results. Dashed lines are theoretical predictions. **d,** The measured transmission of constructive (T_{cons}) and destructive (T_{des}), and the calculated phase bias φ_{bias}. The maximum phase bias φ_{bias} is around 0.3π.

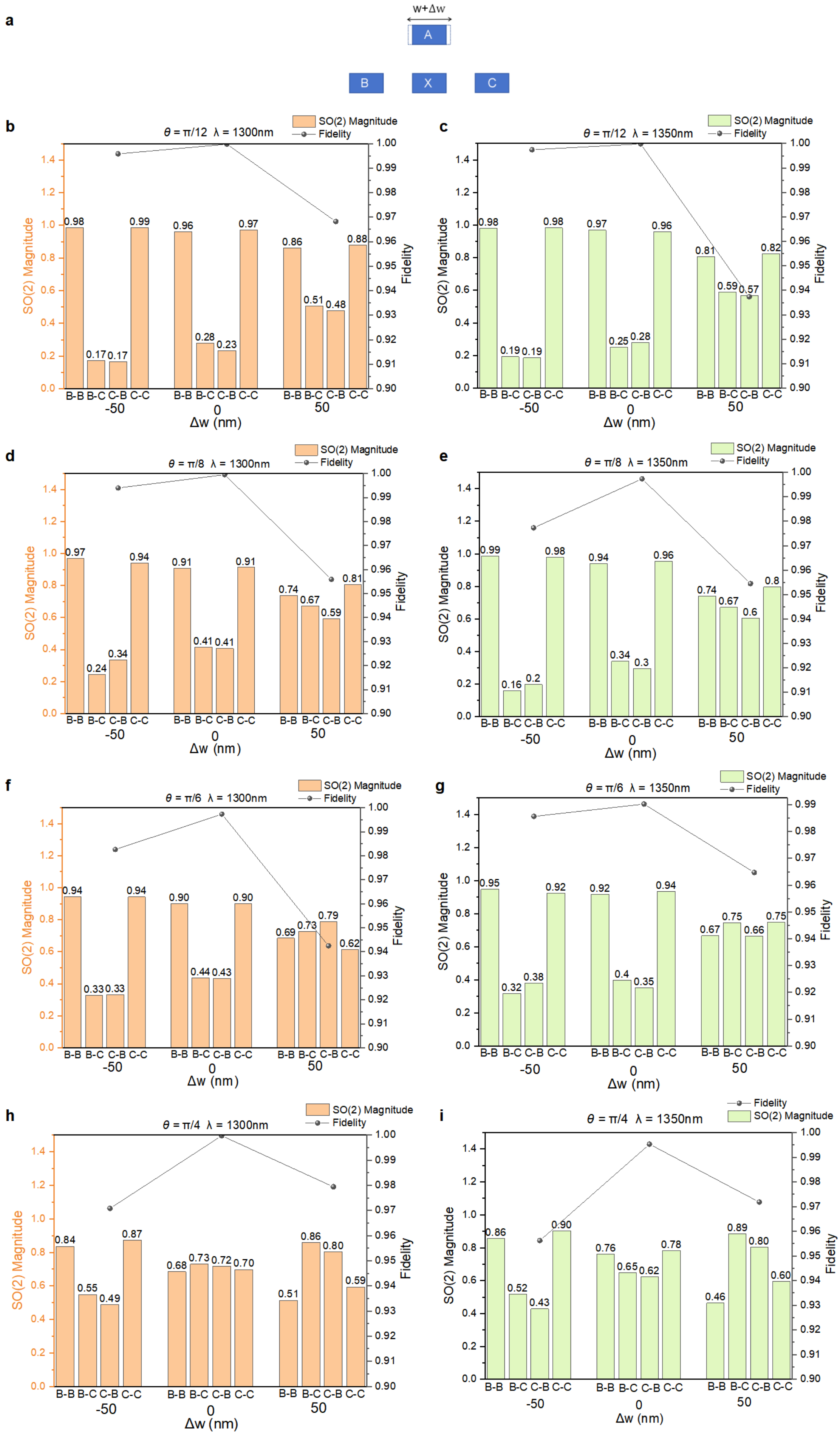

a
w+Δw
A
B
X
C
b
θ = π/12 λ = 1300nm
SO(2) Magnitude
Fidelity
SO(2) Magnitude
Fidelity
0.98 0.17 0.17 0.99 0.96 0.28 0.23 0.97 0.86 0.51 0.48 0.88
B-B B-C C-B C-C
-50 0 50
Δw (nm)
c
θ = π/12 λ = 1350nm
0.98 0.19 0.19 0.98 0.97 0.25 0.28 0.96 0.81 0.59 0.57 0.82
d
θ = π/8 λ = 1300nm
0.97 0.24 0.34 0.94 0.91 0.41 0.41 0.91 0.74 0.67 0.59 0.81
e
θ = π/8 λ = 1350nm
0.99 0.16 0.2 0.98 0.94 0.34 0.3 0.96 0.74 0.67 0.6 0.8
f
θ = π/6 λ = 1300nm
0.94 0.33 0.33 0.94 0.90 0.44 0.43 0.90 0.69 0.73 0.79 0.62
g
θ = π/6 λ = 1350nm
0.95 0.32 0.38 0.92 0.92 0.4 0.35 0.94 0.67 0.75 0.66 0.75
h
θ = π/4 λ = 1300nm
0.84 0.55 0.49 0.87 0.68 0.73 0.72 0.70 0.51 0.86 0.80 0.59
i
θ = π/4 λ = 1350nm
0.86 0.52 0.43 0.90 0.76 0.65 0.62 0.78 0.46 0.89 0.80 0.60

Supplementary Fig. 23| Experimental results of deliberately induce width mismatch in layer 2 of different SO(2). a, In fabrication we induce width-mismatch (±50 nm) between two layers in the whole holonomy. **b-i,** Experimentally measured fidelity, elements' magnitude for different SO(2) at wavelength 1300 nm and 1350 nm as the width of waveguide (layer 2) varies. **b,** $\theta=\pi/12$, at wavelength 1300 nm. **c,** $\theta=\pi/12$, at wavelength 1350 nm. **d,** $\theta=\pi/8$, at wavelength 1300 nm. **e,** $\theta=\pi/8$, at wavelength 1350 nm. **f,** $\theta=\pi/6$, at wavelength 1300 nm. **g,** $\pi/6$, at wavelength 1350 nm. **h,** $\theta=\pi/4$, at wavelength 1300 nm. **i,** $\pi/4$, at wavelength 1350 nm.

a

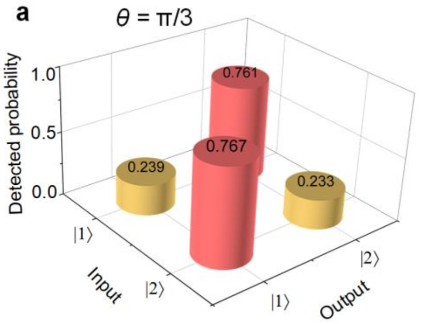

Detection probability

Input \ Output	|1⟩	|2⟩
|1⟩	(23.9±0.3)%	(76.1±0.6)%
|2⟩	(76.7±0.6)%	(23.3±0.2)%

Coincidences in 10 s

Input \ Output	|1⟩	|2⟩
|1⟩	25071	79803
|2⟩	89338	27145

b

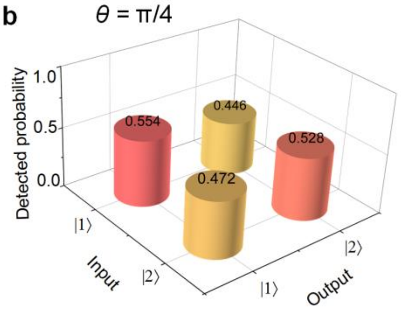

Detection probability

Input \ Output	|1⟩	|2⟩
|1⟩	(55.4±0.2)%	(45.6±0.1)%
|2⟩	(47.2±0.1)%	(52.8±0.2)%

Coincidences in 10 s

Input \ Output	|1⟩	|2⟩
|1⟩	471135	378703
|2⟩	388474	433835

c

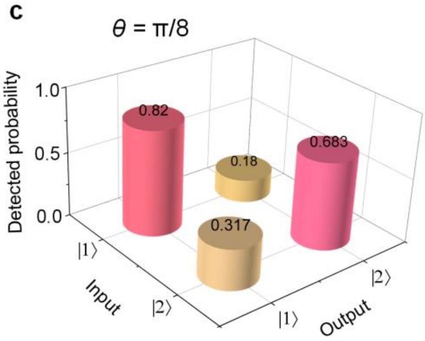

Detection probability

Input \ Output	|1⟩	|2⟩
|1⟩	(82.0±0.5)%	(18.0±0.2)%
|2⟩	(31.7±0.2)%	(68.3±0.4)%

Coincidences in 10 s

Input \ Output	|1⟩	|2⟩
|1⟩	145586	31969
|2⟩	55616	120019

Supplementary Fig. 24. Quantum experimental results of SO(2). The detected probability, measurement error, and coincidences for different elements of SO(2). **a,** SO(2) with $\theta = \pi/3$. **b,** SO(2) with $\theta = \pi/4$. **c,** SO(2) with $\theta = \pi/8$.

a

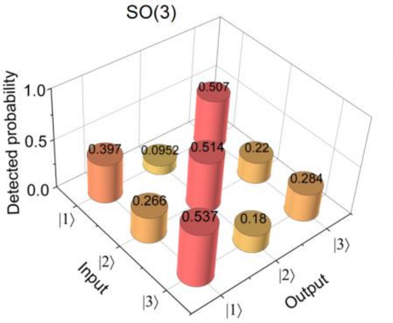

Detection probability

Input \ Output	|1⟩	|2⟩	|3⟩
|1⟩	(39.7±0.3)%	(9.5±0.6)%	(50.7±0.1)%
|2⟩	(26.6±0.3)%	(51.4±0.5)%	(22.0±0.3)%
|3⟩	(53.7±0.5)%	(18.0±0.2)%	(28.4±0.3)%

Coincidences in 10 s

Input \ Output	|1⟩	|2⟩	|3⟩
|1⟩	66183	15863	84526
|2⟩	29930	57778	24761
|3⟩	67309	22520	35608

b

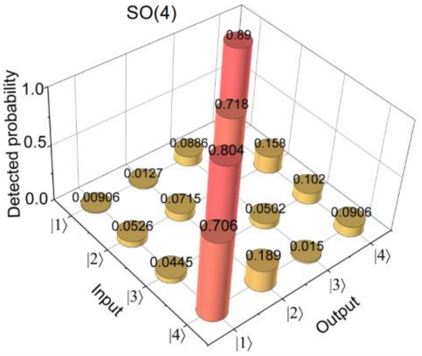

Detection probability

Input \ Output	|1⟩	|2⟩	|3⟩	|4⟩
|1⟩	(0.91±0.03)%	(1.27±0.04)%	(8.9±0.1)%	(89.0±0.7)%
|2⟩	(5.3±0.1)%	(7.1±0.1)%	(71.8±0.6)%	(15.8±0.2)%
|3⟩	(4.4±0.2)%	(80.4±1.3)%	(5.0±0.2)%	(10.2±0.3)%
|4⟩	(70.6±0.9)%	(18.8±0.4)%	(1.5±0.1)%	(9.1±0.2)%

Supplementary Fig. 25. Quantum experimental results of SO(3) and SO(4). a, The detected probability, measurement error, and coincidences of SO(3). **b,** The detected probability, measurement error, and coincidences of SO(4).

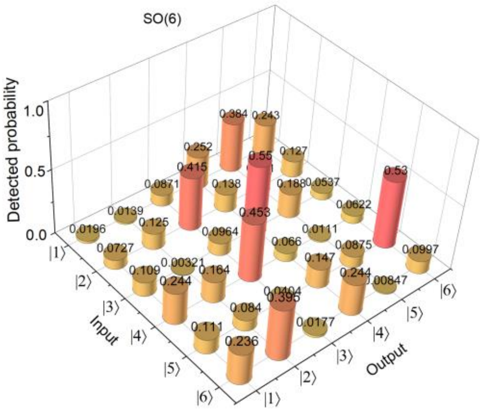

Detection probability

Output / Input	|1⟩	|2⟩	|3⟩	|4⟩	|5⟩	|6⟩
|1⟩	(1.9±0.1)%	(1.4±0.1)%	(8.7±0.3)%	(25.2±0.6)%	(38.4±0.8)%	(24.3±0.6)%
|2⟩	(7.3±0.3)%	(12.5±0.4)%	(41.5±1.0)%	(13.8±0.4)%	(12.1±0.4)%	(12.7±0.4)%
|3⟩	(10.9±0.3)%	(0.32±0.04)%	(9.6±0.3)%	(55.0±1.1)%	(18.8±0.5)%	(5.4±0.2)%
|4⟩	(24.4±0.4)%	(16.4±0.3)%	(45.3±0.7)%	(6.6±0.2)%	(1.1±0.1)%	(6.2±0.2)%
|5⟩	(11.1±0.3)%	(8.4±0.3)%	(4.0±0.2)%	(14.7±0.4)%	(8.8±0.3)%	(53.0±1.1)%
|6⟩	(23.6±0.7)%	(39.5±1)%	(1.8±0.1)%	(24.4±0.7)%	(0.8±0.1)%	(10.0±0.4)%

Supplementary Fig. 26. Quantum experimental results of SO(6). The detected probability and measurement error of SO(6). The experiment is performed through single-photon injection to the 6 input and 6 output device.

a

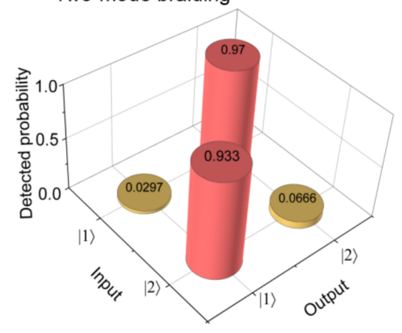

Detection probability

Input \ Output	$\|1\rangle$	$\|2\rangle$
$\|1\rangle$	(3.00±0.06)%	(97.0±0.6)%
$\|2\rangle$	(93.3±0.8)%	(6.7±0.1)%

Coincidences in 10 s

Input \ Output	$\|1\rangle$	$\|2\rangle$
$\|1\rangle$	3328	108723
$\|2\rangle$	64265	4582

b

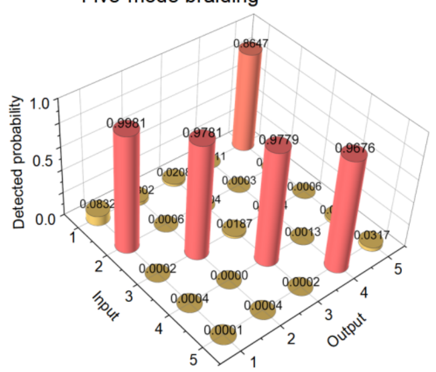

Detection probability

Input \ Output	$\|1\rangle$	$\|2\rangle$	$\|3\rangle$	$\|4\rangle$	$\|5\rangle$
$\|1\rangle$	(8.3±0.3)%	(3.0±0.2)%	(2.0±0.1)%	(1.11±0.01)‰	(86.5±1.9)%
$\|2\rangle$	(99.8±1.5)%	(0.06±0.02)%	(0.04±0.01)%	(0.350±0.003)‰	(0.05±0.02)%
$\|3\rangle$	(0.02±0.01)%	(97.8±1.1)%	(0.2±0.1)%	(2.41±0.01)‰	(0.06±0.01)%
$\|4\rangle$	(0.04±0.01)%	(0.003±0.003)%	(97.8±1.2)%	(1.27±0.01)‰	(2.0±0.1)%
$\|5\rangle$	(0.01±0.01)%	(0.04±0.01)%	(0.02±0.01)%	(96.8±0.6)%	(3.1±0.1)%

Supplementary Fig. 27. Quantum experimental results of braidings. a, The detected probability, measurement error, and coincidences of two-mode braiding. **b,** The detected probability and measurement error of five-mode braiding.

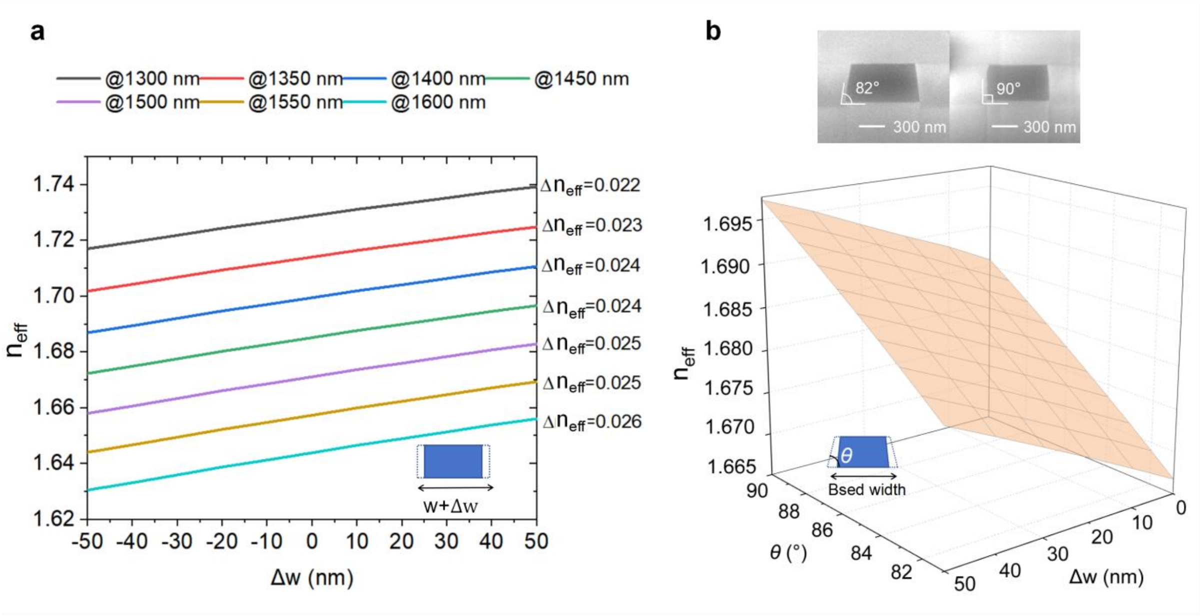

Supplementary Fig. 28| Effective index variation with the change of waveguide width. a, Simulated the waveguide's effective indices as the width variation in ±50 nm at wavelength from 1300 nm to 1600 nm. Δn_{eff} that range from 0.022 to 0.026 are observed. **b,** The inset shows the FIB images of waveguides' cross-sections with different recipes and machines. The illustration below shows the simulated effective indices as angle and width vary at wavelength 1450nm.

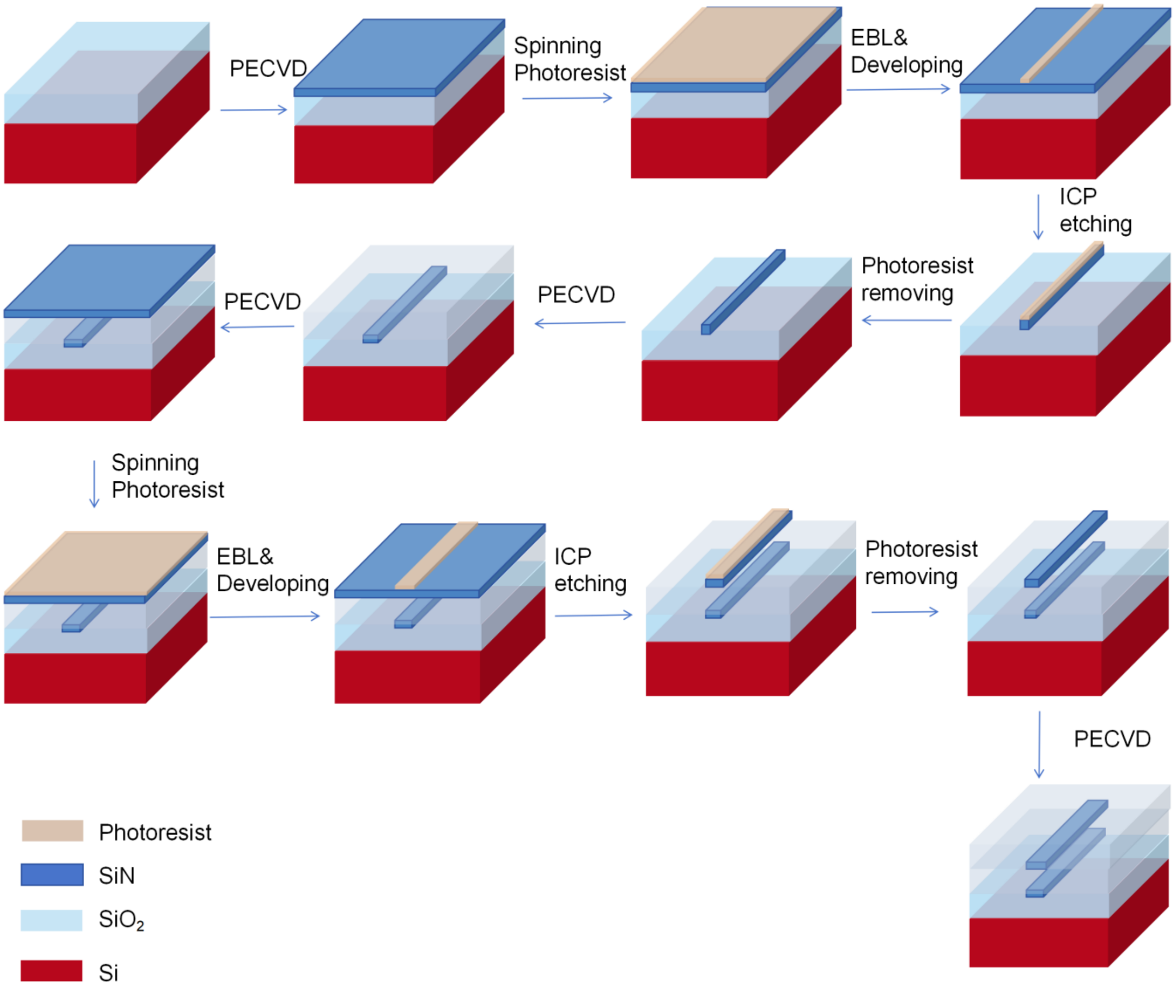

Supplementary Fig. 29| Fabrication process of non-Abelian holonomy device on two-layer SNOI integrated photonic platform. The devices were fabricated on a 500-μm-thick silicon substrate wafer with a 3-μm-thick buried oxide layer. For the first layer, a 450-nm-thick silicon nitride film was deposited by PECVD. Then the photoresist was spun and baked. The designed patterns were defined by electronic beam lithography, and the developing used methyl isobutyl ketone and isopropanol. Then it was fully etched through inductively coupled plasma reactive ion etching. After that, the chip was coated with silica as inter-layer dielectrics using PECVD. For the second layer, similar processes were repeated as the first layer, such as depositing 450-nm-thick silicon nitride film, defining patterns through EBL, fully ICP etching, etc. Finally, a 3 μm-thick silica cladding was deposited through PECVD.

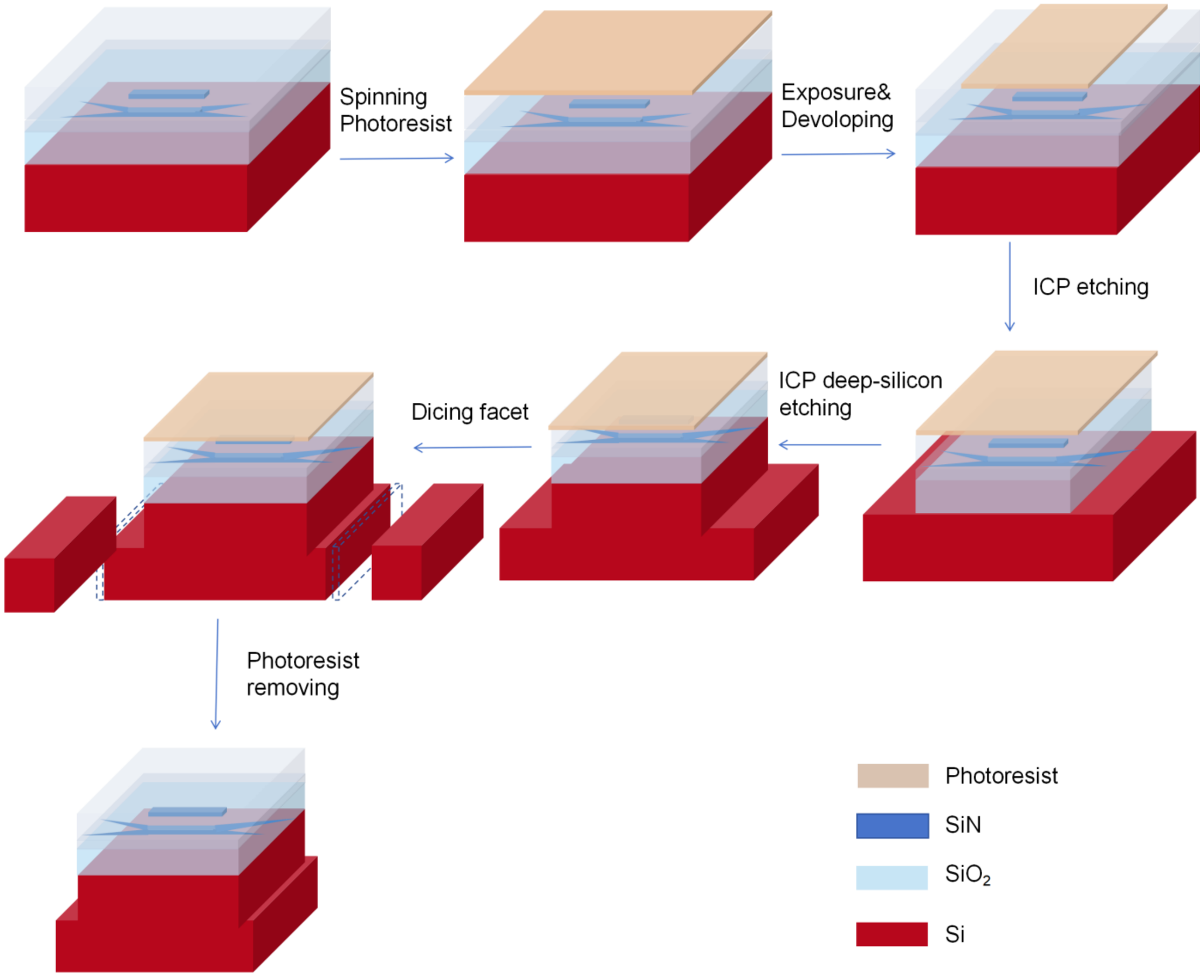

Supplementary Fig. 30| Fabrication process of exposure edge coupler. Firstly, the double-sided alignment contact UV-lithography was used to define patterns that protected devices while exposing the region outside the edge couplers. Then, ICP etching was employed to etch dielectrics until the silicon substrate was exposed. After that, ICP deep-silicon etching was carried out to etch the silicon substrate about 100 μm. Finally, the die sawing system was used to slice the chip.

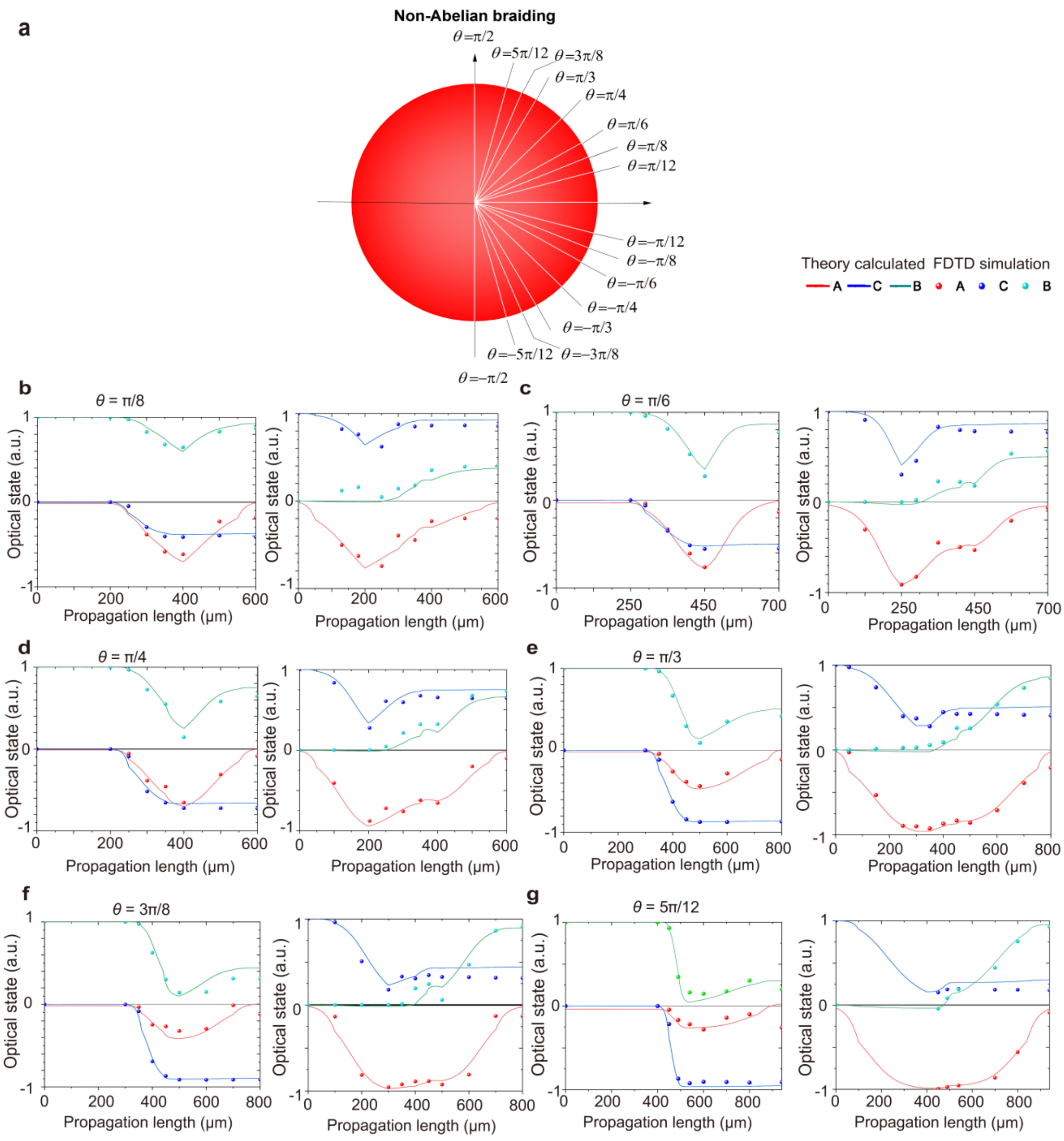

Supplementary Fig. 31| Simulation results through mathematical calculation and FDTD simulation. a, The simulated targeted θ are π/8, π/6, π/4, π/3, 5π/8, 5π/12, respectively. The negative θ is the time-reversal version of the positive θ. **b-g,** For each simulated targeted θ, the left figure is the light injected from waveguide B while the right figure is the light injected from waveguide C. The solid lines represent the theoretically calculated optical state in whole propagation. The dots represent the optical magnitude obtained from the 3D FDTD simulation through monitors at different positions of devices. The corresponding g_{AX}, g_{BX}, g_{CX} and κ_{AX}, κ_{BX}, κ_{CX} in whole evolution, the mathematical integral of Wilczek-Zee connection at wavelength from 1300 nm to 1500 nm, output magnitude form 3D FDTD simulation are shown in Supplementary Figs. 2-3.

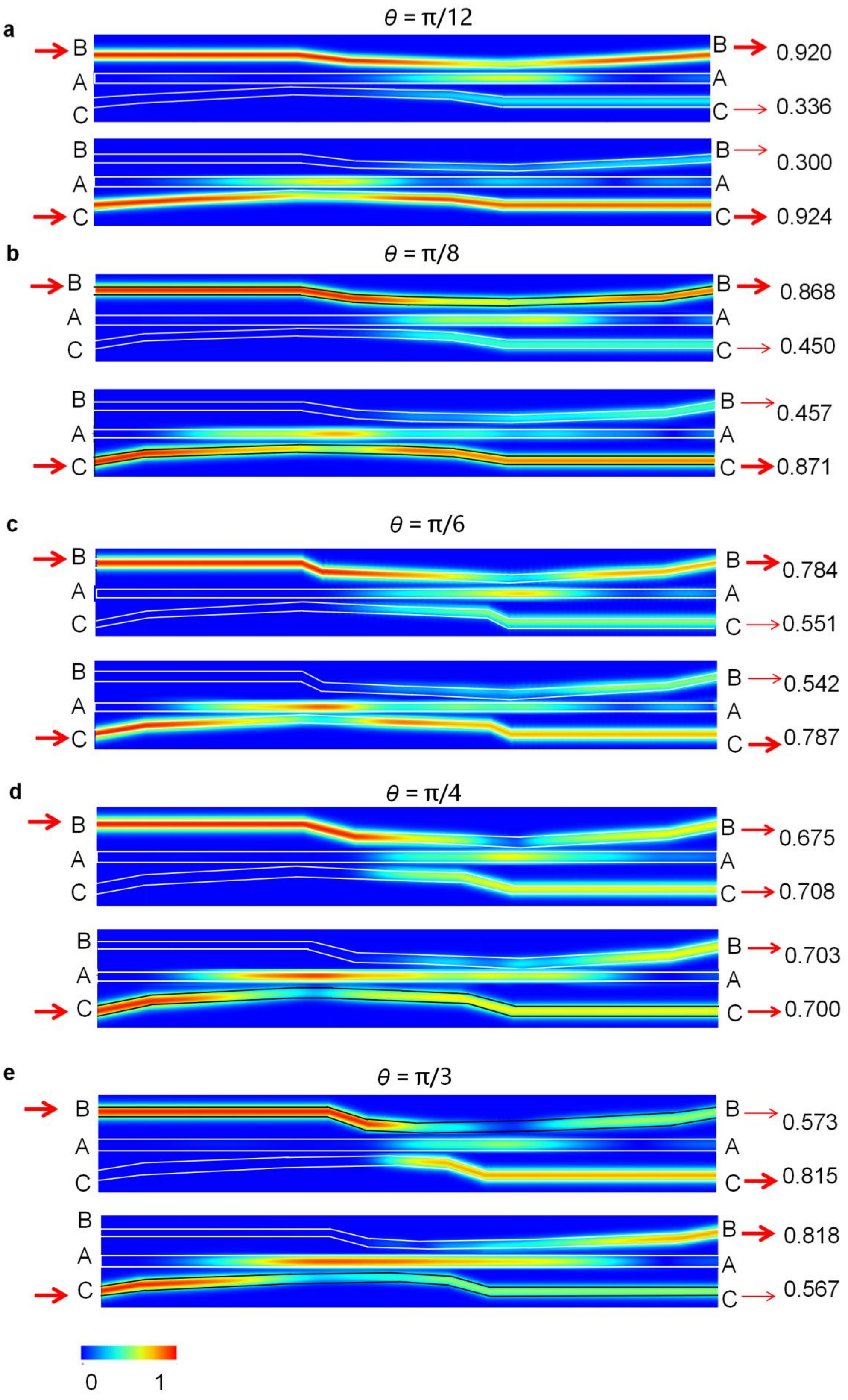

Supplementary Fig. 32| Simulation of optical field distribution in waveguides A, B, C (where the degenerate eigenstates lie in) for different θ**.** The magnitude of the

electromagnetic field in the whole evolution is shown. **a,** θ=π/12. **b,** θ=π/8. **c,** θ=π/6. **d,** θ=π/4. **e,** θ=π/3.

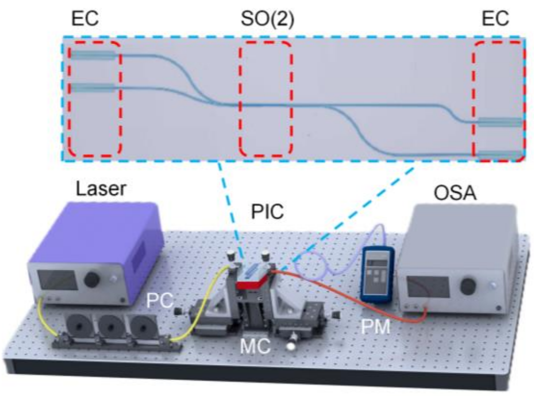

Supplementary Fig. 33| Classical experiment setup and microscope picture of the photonic integrated chip. PC, polarization controller. PIC, photonic integrated circuit. MC, motion controller. PM, power meter. OSA, optical spectrum analyzer. Light is supplied by a wideband source (laser), then it propagates through a polarization controller and is interfaced in/out to the photonic integrated circuit through edge couplers. The output light is monitored by the power meter and analyzed by the optical spectrum analyzer.

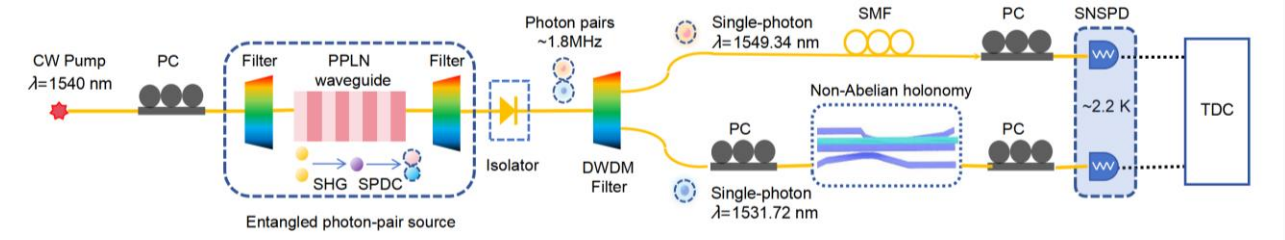

Supplementary Fig. 34| Quantum experiments setups. SHG, second-harmonic generation. SPDC, spontaneous parametric down-conversion. PPLN, fiber pigtailed periodically poled $LiNbO_3$. PC, polarization controller. TDC, time-to-digital converter. SNSPD, superconducting nanowire single-photon detectors. SMP, single-mode fiber. DWDM, dense wavelength division multiplexing. CW, continuous wave. More details of the entangled photon pairs source can refer to Supplementary Reference 5**.**

References

1. Neef, V. et al. Three-dimensional non-Abelian quantum holonomy. *Nat. Phys.* **19**, 30–34 (2023).
2. Kremer, M., Teuber, L., Szameit, A. & Scheel, S. Optimal design strategy for non-Abelian geometric phases using Abelian gauge fields based on quantum metric. *Phys. Rev. Res.* **1**, 033117 (2019).
3. Hunsperger, R. G. *Integrated Optics: Theory and Technology* (Springer, Berlin, 2010).
4. Pinske, J. & Scheel, S. Symmetry-protected non-Abelian geometric phases in optical waveguides with nonorthogonal modes. *Phys. Rev. A* **105**, 013507 (2021).
5. Zhang, Z. et al. High-performance quantum entanglement generation via cascaded second-order nonlinear processes. *npj Quantum Inf.* **7**, 123 (2021).
